# Stepwise conversion of the Cys_6_[4Fe–3S] to a Cys_4_[4Fe–4S] cluster and its impact on the oxygen tolerance of [NiFe]-hydrogenase†

**DOI:** 10.1039/d3sc03739h

**Published:** 2023-09-20

**Authors:** Andrea Schmidt, Jacqueline Kalms, Christian Lorent, Sagie Katz, Stefan Frielingsdorf, Rhiannon M. Evans, Johannes Fritsch, Elisabeth Siebert, Christian Teutloff, Fraser A. Armstrong, Ingo Zebger, Oliver Lenz, Patrick Scheerer

**Affiliations:** a Charité – Universitätsmedizin Berlin, Corporate Member of Freie Universität Berlin and Humboldt-Universität zu Berlin, Institute of Medical Physics and Biophysics (CC2), Group Structural Biology of Cellular Signaling Charitéplatz 1 10117 Berlin Germany patrick.scheerer@charite.de; b Institut für Chemie, Biophysical Chemistry, Technische Universität Berlin Straße des 17. Juni 135 10623 Berlin Germany oliver.lenz@tu-berlin.de ingo.zebger@tu-berlin.de; c Department of Chemistry, University of Oxford OX1 3QR Oxford UK; d Department of Physics, Freie Universität Berlin Arnimallee 14 14195 Berlin Germany

## Abstract

The membrane-bound [NiFe]-hydrogenase of *Cupriavidus necator* is a rare example of a truly O_2_-tolerant hydrogenase. It catalyzes the oxidation of H_2_ into 2e^−^ and 2H^+^ in the presence of high O_2_ concentrations. This characteristic trait is intimately linked to the unique Cys_6_[4Fe–3S] cluster located in the proximal position to the catalytic center and coordinated by six cysteine residues. Two of these cysteines play an essential role in redox-dependent cluster plasticity, which bestows the cofactor with the capacity to mediate two redox transitions at physiological potentials. Here, we investigated the individual roles of the two additional cysteines by replacing them individually as well as simultaneously with glycine. The crystal structures of the corresponding MBH variants revealed the presence of Cys_5_[4Fe–4S] or Cys_4_[4Fe–4S] clusters of different architecture. The protein X-ray crystallography results were correlated with accompanying biochemical, spectroscopic and electrochemical data. The exchanges resulted in a diminished O_2_ tolerance of all MBH variants, which was attributed to the fact that the modified proximal clusters mediated only one redox transition. The previously proposed O_2_ protection mechanism that detoxifies O_2_ to H_2_O using four protons and four electrons supplied by the cofactor infrastructure, is extended by our results, which suggest efficient shutdown of enzyme function by formation of a hydroxy ligand in the active site that protects the enzyme from O_2_ binding under electron-deficient conditions.

## Abstract 2

The [4Fe-3S] cluster of an O_2_-tolerant [NiFe] hydrogenase was transformed into different [4Fe-4S] clusters, and structural, spectroscopic and electrochemical analyses of the enzyme variants revealed an O_2_-tolerance mechanism at various levels.

## Graphical abstract

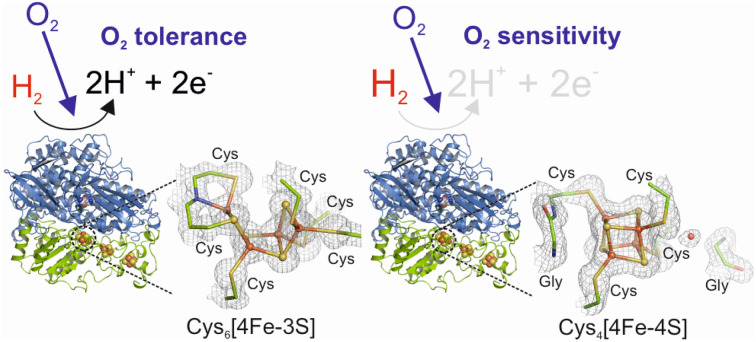

## Introduction

**Fig. 1:**
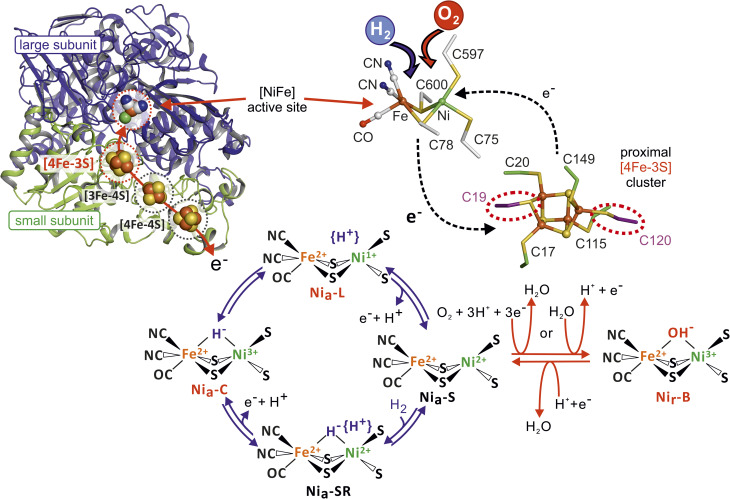
Overview of oxygen-tolerant membrane-bound [NiFe] hydrogenase from *Cupriavidus necator* (MBH). Left: Cartoon representation of the MBH heterodimer (large subunit HoxG in blue, small subunit HoxK in green) in the reduced state (PDB entry 3RGW). The [NiFe] active site in the large subunit and the proximal [4Fe–3S] cluster (both highlighted by dashed red circles) as well as the remaining Fe–S clusters of the small subunit (marked by dashed black circles) are shown in a ball representation. The red arrow illustrates the electron pathway between the active site and the iron–sulfur clusters. Right: Close up view of the [NiFe]-active site and the unique [4Fe–3S] cluster with its two supernumerary cysteines (dashed red circles). Depending on the presence of oxygen or hydrogen, the electron exchange between the two metal-centers occurs in a different direction. Bottom: Illustration of the various redox states of the [NiFe]-active site during catalytic H_2_ oxidation/proton reduction (blue arrows) and the proposed in-/re-activation process (red arrows) upon O_2_ exposure. Active site states (Ni_u_-A, Ni_ia_-S), which are discussed in the text but whose structures are unknown and presumably formed only under *in vitro* conditions are not shown.

Molecular hydrogen has been almost inexhaustible on the ancient earth and is supposed to represent one of the first energy sources for early microbes.^1^ Biocatalytic splitting of H_2_ into protons and electrons occurs at transition metal centers that constitute the active sites of hydrogenases. Depending on the metal center composition, these metalloenzymes are classified as either [FeFe]- or [NiFe]-hydrogenases.^2,3^ Consistent with their anaerobic origin, hydrogenases are commonly highly susceptible to molecular oxygen, which either inactivates or even destroys the enzymes. Thus, the emergence of the oxygenic atmosphere posed evolutionary pressure towards a microbial metabolism that also functions under oxic conditions. Although the availability of free H_2_ declined dramatically during these times, some bacterial species evolved O_2_-tolerant [NiFe]-hydrogenases that feed the H_2_-derived electrons into the quinone pool of the aerobic respiratory chain.^4^ A prominent member of these so-called *Knallgas* bacteria is *Cupriavidus necator* H16 (formerly *Ralstonia eutropha* H16), which employs four different O_2_-tolerant [NiFe]-hydrogenases for generating cellular energy from the controlled oxidation of H_2_ in the presence of O_2_.^5^ Among them, only the tripartite membrane-bound [NiFe]-hydrogenase (MBH) is directly linked to the respiratory chain.^6^ The MBH large subunit contains the catalytic center composed of a nickel and an iron ion, which are coordinated to the protein by four Cys-derived thiolates. One CO and two cyanide ligands additionally ligate the iron (Fig. 1). This architecture provides a substrate/inhibitor-binding site located between the two metal ions.^7,8^ Notably, the first and second coordination spheres of the MBH catalytic center are identical to those of O_2_-sensitive [NiFe]-hydrogenases, suggesting that the O_2_ tolerance of the enzyme originates from structural features furtheraway from the active site.^9^ Besides variations in their gas tunnel networks,^10,11^ a major difference in cofactor structure/composition has been found in the small, electron-transferring subunit of the *C. necator* MBH and closely related enzymes compared to O_2_-sensitive hydrogenases.^9,12^ Typically, membrane-bound [NiFe]-hydrogenases contain a relay of three iron–sulfur clusters that connect the active site with the third subunit, a cytochrome *b*. The most distal cluster relative to the active site is a [4Fe–4S] cluster coordinated by three cysteines and a histidine, while the medial cluster is a [3Fe–4S] species with three cysteines as protein anchors.^2,3^ However, in the proximal position, instead of a standard Cys_4_[4Fe–4S] cluster, the O_2_-tolerant MBHs possess a [4Fe–3S] cluster coordinated by six cysteine residues (Fig. 1 and S1†).^13–16^ The unique architecture of the [4Fe–3S] cluster is associated with a redox-dependent structural flexibility that allows two redox-transitions within a physiological potential region, *i.e.*, the cofactor can assume a super-oxidized, an oxidized, and a reduced state.^13,15,17^

This unusual trait is supported by a plethora of biochemical, electrochemical, spectroscopic, computational, and crystallographic results obtained for MBH enzymes from *Aquifex aeolicus*, *Escherichia coli*, *Hydrogenovibrio marinus*, *Cupriavidus necator*, and *Salmonella enterica*.^12–17^ Moreover, a functional [4Fe–3S] cluster is essential for O_2_ tolerance, as the replacement of both or just one of the two additional cysteine residues results in the loss of the capability to sustain H_2_ oxidation at high O_2_ concentrations. This requirement has been demonstrated for the MBH from *C. necator* and the closely related Hyd-1 from *E. coli* based on bacterial growth and protein film electrochemistry (PFE) experiments.^9^ According to a current model, the proximal [4Fe–3S] cluster contributes to efficient O_2_ detoxification in the catalytic center, *i.e.*, rapid and complete reduction of O_2_ to 2H_2_O with 4e^−^ and 4H^+^ previously obtained from H_2_ oxidation (Fig. 1).^9,15^ In fact, the production of water as a result of O_2_ reduction with H_2_-derived electrons has already been reported for another O_2_-tolerant [NiFe]-hydrogenase from *C. necator*^18^ and Hyd-1 from *E. coli*.^19^ Catalytic intermediates of the H_2_/H^+^ cycling process at [NiFe]-hydrogenase active sites are well-established (Fig. 1). Notably, the only well-defined, oxygen-containing intermediate is an active site featuring Ni^3+^ and Fe^2+^ ions bridged by an OH^−^ ligand.^3^ This so-called Ni_r_-B state is considered to represent a resting state of the enzyme that is rapidly re-activated upon reduction, although there is no experimental evidence that O_2_ is the origin of the bridging hydroxy ligand.^20,21^ Further O_2_-mediated modifications of the catalytic center, which could also be caused by X-ray damage, include oxygenated cysteines observed in a number of crystal structures of both O_2_-sensitive and O_2_-tolerant [NiFe]-hydrogenases.^13,15,22–24^ Such oxygenated cysteine thiolates are considered to impede catalytic activity either temporarily or even permanently, and are associated with the inactive state referred to as Ni_u_-A (catalytically defined as an ‘unready’ state).^3,22,25^

Prevention of any oxidative damage of the active site is a prerequisite for sustained H_2_ oxidation activity under aerobic conditions, and the proximal [4Fe–3S] cluster appears to play a crucial role in this protective mechanism. In this context, *C. necator* MBH variants bearing cysteine exchanges at the proximal [4Fe–3S] cluster were analyzed under various oxidizing and reducing conditions by X-ray crystallography, biochemistry, protein film electrochemistry, spectroscopy, and spectro-electrochemistry to investigate the role of the two supernumerary cysteines at the proximal cluster. The results are of great importance for a generalization on the origin of O_2_-tolerance of [NiFe]-hydrogenases.

## Results

In order to obtain insight into the individual role of the supernumerary cysteines in the coordination and function of the [4Fe–3S] cluster, we purified *C. necator* MBH derivatives carrying exchanges of cysteine for glycine at the positions 19 and/or 120 in the small subunit HoxK, which are hereafter referred to as MBH^C19G^, MBH^C120G^ and MBH^C19G/C120G^. While the native MBH and the MBH^C19G^ protein variants showed similar yields, approx. half the amount was obtained for MBH^C19G/C120G^ (Table S1†). Only a fractional amount of pure protein per g of cells was collected for MBH^C120G^. The H_2_-driven methylene blue reduction activities of the purified MBH^C19G^, MBH^C120G^ and MBH^C19G/C120G^ proteins were 53%, 13%, and 52%, respectively, of the activity of native MBH (140 U mg^−1^ ref. 9) (Table S1†).

To obtain a detailed view of the proximal cluster architecture, all three engineered MBH variants were analyzed by protein X-ray crystallography. Additionally, the two MBH^C19G^ and MBH^C120G^ variants were investigated by PFE and surface-enhanced infrared absorption (SEIRA) spectro-electrochemistry as well as infrared (IR), resonance Raman (RR) and electron paramagnetic resonance (EPR) spectroscopy, to correlate activities with the structural/electronic properties of the metal centers.

### Cysteine exchanges lead to major changes in the composition and architecture of the proximal Fe–S cluster

The MBH^C19G^, MBH^C120G^ and MBH^C19G/C120G^ variants were crystallized under either aerobic/oxidizing or anaerobic/H_2_-reducing conditions. The crystal structures were solved by molecular replacement, and the models were refined to resolutions ranging from 1.60 Å to 1.93 Å (PDB entries 8POU-8POZ). To qualify positive peaks in the *mF*_*o*_*-DF*_*C*_ electron density maps, anomalous diffraction data were collected.

#### MBH^C19G^

**Fig. 2:**
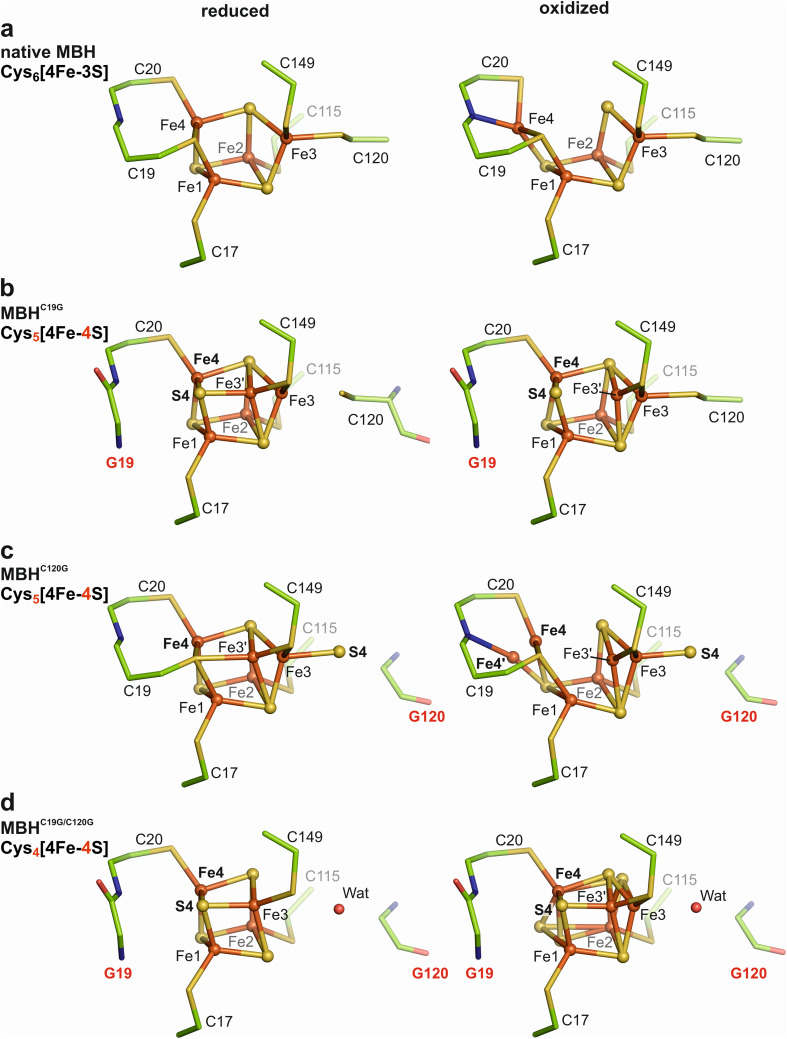
Structures of the proximal iron–sulfur clusters of crystals of native MBH and variants grown under H_2_-reducing and oxidizing conditions. The proximal clusters of (a) native MBH, (b) MBH^C19G^, (c) MBH^C120G^, and (d) MBH^C19G/C120G^ are depicted in ball/stick representation. Iron (Fe), sulfur (S), carbon (C), oxygen (O), and nitrogen (N) atoms are shown as orange, yellow, green, red, and blue sticks and/or spheres, respectively. Water molecules (wat) are illustrated as red spheres. Numbering of amino acid and cluster ions refers to native MBH.

Crystals of oxidized and H_2_-reduced MBH^C19G^ diffracted to resolutions up to 1.61 Å and 1.60 Å, respectively (Table S2†). H_2_-reduced MBH^C19G^ crystals harbored an almost cubic [4Fe–4S] cluster in the proximal position (Fig. 2b). In contrast to the native [4Fe–3S] cluster (Fig. 2a), the bridging function of the Cys19-derived thiolate was replaced with a sulfide (S4) coordinating both Fe1 and Fe4. Surprisingly, the Fe3 coordination to Cys120 was essentially lost. According to the electron density maps (Fig. S2†) only *ca.* 20% of Fe3 remained in the native-like (“classical”) position, previously observed for the Fe3 in the native [4Fe–3S] cluster. Approximately 80% of Fe3 were shifted by 1.4 Å to a position corresponding to a cubane corner (Fe3′), thereby forming a bond to S4 instead of Cys120 (Fig. 2b). In fully oxidized MBH^C19G^, a different ratio of the Fe3 positions was observed. Approximately 70% of Fe3 remained coordinated by Cys120, and only 30% was found in the Fe3′ position (Fig. 2b). The corresponding 2*mF*_*o*_*-DF*_*C*_ electron density map (Fig. S2†) indicated no covalent bond between Fe3′ and S4 (distance of 2.7 Å). Notably, the Fe4 did not change its position (in contrast to the super-oxidized state of the proximal cluster in native MBH), which is in agreement with the absence of the Cys19-derived thiolate (Fig. 2b and S2†).

#### MBH^C120G^

Replacing cysteine 120 with glycine yielded crystals of H_2_-reduced MBH^C120G^ that diffracted to a resolution of 1.65 Å (Table S2†). The *mF*_*o*_*-DF*_*c*_ electron density map can be interpreted as a [4Fe–3S] cluster with an additional positive density at the former position of the Cys120 thiolate (Fig. 2c and S3†). Single-wavelength anomalous dispersion (SAD) data suggest that the positive density corresponds to an Fe3-coordinating sulfur species (most likely S^2−^ or SH^−^) (Fig. S3d†). It should be noted that the presence of a chloride ion is generally also possible at this position. Despite having a standard [4Fe–4S] composition, the cluster displayed an unusual architecture carrying an exo-sulfur (S4) (Fig. 2c). As in MBH^C19G^, Fe3 occurs in a double conformation, but always retained a tetrahedral coordination. Approximately 60% of the Fe3 resided within bonding distance to the exo-sulfur. The remaining 40% (Fe3′) were located at 2.5 Å distance from the thiolate group of Cys19, which was also at a bond distance to Fe1 and Fe4 (Cys19-Sγ-Fe1 distance 2.3 Å, Cys19-Sγ-Fe4 distance 2.4 Å, Fig. 2c).

The electron density maps (Fig. S3†) of oxidized MBH^C120G^ crystals (at 1.93 Å resolution) revealed the proximal Fe–S cluster in a similar structure/composition as in the reduced protein, except that Fe4 showed conformational flexibility (Fig. 2c). The electron density distribution was interpreted with 50% of Fe4 residing in a comparable position as in H_2_-reduced MBH^C120G^ (Fig. 2c). However, an Fe4–S3 distance of 2.9 Å indicates the absence of a covalent bond (Fig. 2c). The remaining 50% of Fe4′ in oxidized MBH^C120G^ were shifted by 1.2 Å to form a covalent bond with the backbone nitrogen of Cys20, bringing Fe4′ into hydrogen bonding distance (2.8 Å) with Glu76 of the MBH small subunit. This particular Fe4′ position differs by 0.6 Å from the classical Fe4 position of native MBH in the super-oxidized state, and the distance between Fe4′ and Sγ-Cys19 and Sγ-Cys20 is 2.9 Å and 2.7 Å, respectively. As observed for all single cluster variants of MBH, the Fe3 was found in a double conformation. Approximately 75% resided in the native Fe3 position and formed a covalent bond to S4 (Fe3–S4 distance 2.4 Å), consistent with the S4 occupancy. The remaining 25% of the Fe3 were shifted by 1.1 Å toward a more cubic conformation (Fe3′). However, a covalent binding to Sγ-Cys19 was not established (Fe3′ – Sγ-Cys19 distance 3.2 Å), resulting in a three-fold ligation of Fe3′ (Fig. S3b†).

#### MBH^C19G/C120G^

The crystal structure of H_2_-reduced MBH^C19G/C120G^ possessed a resolution of 1.92 Å (Table S2†). The corresponding electron density map indicated the presence of a conventional cubic [4Fe–4S] cluster coordinated by four cysteine residues (Fig. 2d, S4 and S5†). The function of the Cys19-derived thiolate bridging Fe1 and Fe4 in the native [4Fe–3S] cluster, was taken over by the additional sulfide S4, which also formed a covalent bond to Fe3. Oxidized crystals of the same variant diffracted with a resolution of up to 1.65 Å (Table S2†). The corresponding crystal structure also showed a “cubane-like” [4Fe–4S] cluster with some structural flexibility of the Fe3, S1 and S3 positions (Fig. 2) compared to the reduced MBH^C19G/C120G^. Interestingly, the *mF*_*o*_*-DF*_*c*_ electron density maps of both oxidized and reduced MBH^C19G/C120G^ uncovered a positive density at the former position of Cys120, which is most likely a water molecule based on SAD data (Fig. 2d and S4d†). The replacement of Cys120 with glycine leads to an increased solvent accessibility of the proximal cluster (Fig. S6†). Notably, the Fe1-bound OH^−^ group of the super-oxidized [4Fe–3S] cluster, which has previously been discovered for native as-isolated MBH,^17^ was not observed in crystal structures of any of the MBH variants.

### Structure of the [NiFe]-active site in the [4Fe–3S] cluster variants

The active site structures of the MBH variants carrying modified proximal iron–sulfur clusters were found in the same conformations as previously described for native MBH (Fig. S7†).^14,17^ The identity includes the positions of the two metal atoms as well as the CO and the two CN^−^ ligands of the iron, which were modelled into the structure according to previous QM/MM calculations.^17^ The oxidized structures exhibited an electron density between the Ni and Fe ions (Ni–Fe) distance, 2.9 Å; (Fig. S7†), which is consistent with the presence of a hydroxyl group occupying the bridging position between the two metals corresponding to the resting state known as Ni_r_-B (Fig. 1). The reduced structures exhibited a lower Ni–Fe distance of 2.6 Å, which is typical of catalytic cycle intermediates^26^ and consistent with our spectroscopic studies (see below). One exception was found for H_2_-reduced MBH^C19G^, which contained a positive *mF*_*o*_*-DF*_*c*_ electron density between nickel and iron (Fig. S7†). We interpreted this density as a mono-oxygen species with an occupancy of about 50%, causing a slightly enlarged Ni–Fe distance of 2.7 Å. A very minor positive electron density located between nickel and iron was also observed for H_2_-reduced MBH^C19G/C120G^. This small density was interpreted as a mono-oxygen species with an occupancy below 10%. Furthermore, the oxidized MBH^C19G^ variant contained a sulfoxygenated (occupancy of 20%), nickel-coordinating cysteine (Cys597). Such modification was less evident in the structure of H_2_-reduced MBH^C19G^ (occupancy below 10%) and can therefore be considered as partly reversible. Notably, we found no evidence of oxygenation at the coordinating cysteine thiolates or of further damage in any of the other crystal structures.

### Spectroscopic properties of the metal cofactors in the MBH variants

To investigate changes in the redox properties for the [NiFe]-active site and the proximal Fe–S cluster of MBH caused by the C19G and C120G exchanges, we carried out IR and EPR spectroscopic experiments in solution, complemented by resonance Raman data obtained from single crystals. Goris *et al.*^9^ already described the IR/EPR-spectroscopic characterization of the MBH^C19G/C120G^ variant, and we will refer to these data mainly in the discussion.

#### MBH^C19G^

**Fig. 3:**
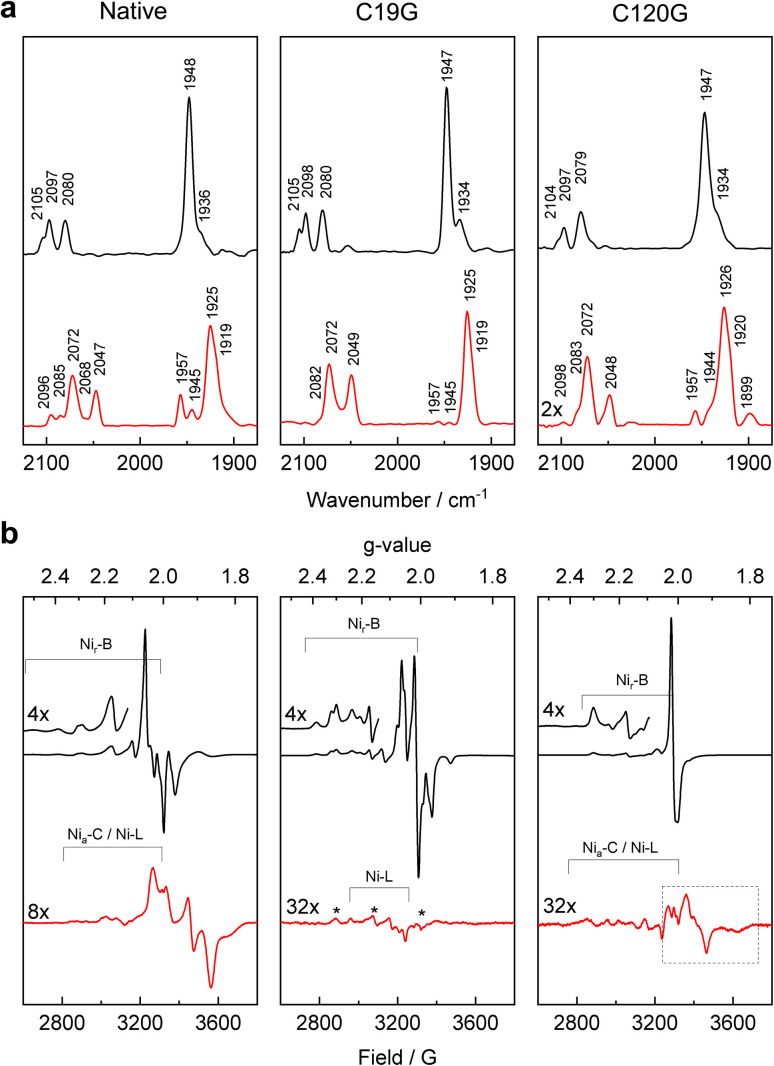
IR and EPR spectroscopic investigation of the MBH variants. (a) IR absorbance spectra of oxidized (black) and H_2_-reduced (red) samples of native MBH (left), MBH^C19G^ (middle), and MBH^C120G^ (right), recorded at 10 °C. The band assignment is summarized in Table S3.† (b) EPR spectra of oxidized (black) and H_2_-reduced (red) samples of native MBH (left), MBH^C19G^ (middle), and MBH^C120G^ (right) measured at 20 K. Signals marked with an asterisk (*g*_*x*_ = 2.31, *g*_*y*_ = 2.15, *g*_*z*_ = 2.00) correspond to a so far unknown paramagnetic state of the active site. The signals in the dashed box can be assigned to different reduced Fe–S clusters (see Fig. S11†).

The IR spectrum of oxidized MBH^C19G^ displayed a similar band pattern for the [NiFe]-active site as observed for native MBH (Fig. 3a), indicating preferential formation of the Ni_r_-B state with characteristic stretching frequencies of 1947 cm^−1^ for the Fe-bound CO as well as 2080 cm^−1^ and 2098 cm^−1^ for the two Fe-ligated CN^−^ ligands. Minor spectral contributions assigned to Ni_r_-S states (Table S3†) were also present.^27^ Both states are consistent with the crystal structure data (Fig. S7†), indicating that the vacant binding position of the [NiFe]-active site is fully occupied with a bridging hydroxy ligand. The corresponding EPR spectrum (at 20 K) revealed a mixture of signals composed of a prominent signal at *g* = 2.01 and a complex coupled signal at *g* = 2.4–1.9 (Fig. 3b). A comparison with the spectrum of oxidized native MBH (Fig. 3b) suggests that the coupled signal originates from the magnetic interaction of the super-oxidized proximal cluster with further paramagnetic centers, *i.e.* the medial [3Fe–4S] cluster and the active site.^9^ In fact, most of the signals occurring in the lower field range (*g* = 2.4–2.0) correspond to the magnetically coupled active site in the Ni_r_-B state (Fig. 3b). The narrow signal around *g* = 2.01 originates from the uncoupled medial [3Fe–4S] cluster. This mixture of spectroscopic signals would be consistent with the structural flexibility of Fe3 (bound to Cys120) and Fe3′ (unbound to Cys120) of the proximal cluster in the oxidized MBH^C19G^ crystal structure (Fig. 2). Indeed, these findings indicate that a significant fraction of the oxidized proximal cluster of the MBH^C19G^ variant resided in a state comparable to that of the paramagnetic, super-oxidized [4Fe–3S] cluster of the native MBH. It is important to mention, however, that the spectral patterns of the coupled signals for both the proximal Fe–S cluster and the active site Ni_r_-B species differ from those of native MBH (Fig. 3b), underpinning the structural differences observed for the proximal Fe–S clusters of native MBH and MBH^C19G^ (Fig. 2). An EPR spectrum recorded at 80 K, in which Fe–S cluster-related signals disappear due to a fast spin relaxation, revealed an uncoupled Ni_r_-B signature (Fig. S8†), indicating that the C19G exchange did not result in the formation of the inactive Ni_u_-A state, commonly found in O_2_-sensitive [NiFe] hydrogenases.^3^

The IR spectrum of the reduced MBH^C19G^ variant (Fig. 3a) was dominated by one of the EPR-silent, fully reduced states, namely Ni_a_-SR′, with characteristic modes at 1925, 2049 and 2072 cm^−1^. A shoulder at 1919 cm^−1^ also indicated the presence of the Ni_a_-SR′′ species (Table S3†). In addition to these states, the corresponding IR spectrum of native MBH contained bands at 1945 and 1957 cm^−1^ (Fig. 3a). This observation implies that a fraction of the active site in native enzyme resides in the Ni_a_-SR state (∼5%) and the paramagnetic Ni_a_-C state (∼10%). The spectrum of the reduced MBH^C19G^ variant showed only traces of these reduced states, suggesting different steady state equilibria caused by the modified proximal cluster.

EPR measurements revealed that H_2_-treated MBH^C19G^ was mainly EPR-silent. Only trace amounts of Ni_a_-L were observed. In contrast, H_2_-reduced native MBH showed a distinct signal typical for the reduced proximal [4Fe–3S] cluster in O_2_-tolerant MBHs from different organisms (Fig. 3b, red traces). It should be noted that clear indications for the reduced distal [4Fe–4S] cluster have not been observed by EPR.^9,28–30^

To investigate whether the reduction of MBH^C19G^ is reversible, we exposed the initially H_2_-reduced enzyme to air. According to EPR, this re-oxidation should have led to a diminishment of both the EPR signal of the proximal cluster and its magnetic coupling to the active site in the Ni_r_-B state (Fig. S9a,† dotted lines). Instead, a large uncoupled axial signal appeared (*g*^⊥^ = 2.017; *g*∥ = 2.001), which was assigned to the oxidized medial [3Fe–4S]^+^ cluster. A similar observation was obtained for native MBH (Fig. S9a†).^9^ The corresponding IR spectrum of re-oxidized MBH^C19G^ protein was dominated by signals related to the Ni_r_-B state (Fig. S9b†), while ∼20% of the protein molecules resided in the inactive Ni_ia_-S state (Table S3†), which should be irreversible under physiological conditions but has eluded structural characterization to date.^29^ Again, native MBH showed a similar behavior (Fig. S9b†). These results suggest that isolated native MBH and MBH^C19G^ suffered from the re-oxidation treatment in the same way. Notably, the oxidative inactivation was completely reversible when the same procedure was performed with membrane fragments (Fig. S9a,† solid lines). This observation shows that the results obtained with the isolated heterodimeric enzyme have to be treated with caution.^29^

In conclusion, the absence of any Fe–S cluster-related EPR signal in the reduced MBH^C19G^ variant can be interpreted as the presence of a diamagnetic proximal cluster. This result suggests that the new Cys_5_[4Fe–4S] cluster in MBH^C19G^ undergoes only one redox transition, corresponding to a reductive conversion from a paramagnetic, super-oxidized state into an EPR-silent oxidized state. The second reduction step towards a fully reduced state appears to be beyond physiological redox potentials. This assumption is supported by the incomplete disappearance of characteristic Fe–S bands (at *ca.* 340 cm^−1^) in the resonance Raman spectrum of H_2_-reduced MBH^C19G^ crystals when compared to the corresponding spectra of native MBH and MBH^C120G^ (Fig. S10†).

#### MBH^C120G^

As with native MBH and MBH^C19G^, the active site of oxidized MBH^C120G^ resided mostly in the Ni_r_-B state, as revealed by IR spectroscopy (Fig. 3a and Table S3†). Consistently, the corresponding EPR spectrum also contained the typical signature of the Ni_r_-B state (Fig. 3b and S8†). Furthermore, the typical axial signal attributable to the oxidized medial [3Fe–4S]^+^ cluster was observed. Notably, both the Ni_r_-B and the medial [3Fe–4S]^+^ cluster signals showed no evidence for magnetic coupling (Fig. 3b), indicating that the oxidized proximal cluster resided in a diamagnetic, EPR-silent state. This result suggests that the proximal [4Fe–4S] cluster of MBH^C120G^ has lost the ability to adopt the super-oxidized state typical of the native proximal [4Fe–3S] cluster^17^ due to the lack of Cys120 binding to Fe3 (Fig. 2c).

The observed bands in the IR spectrum of H_2_-reduced MBH^C120G^ correspond to the catalytically relevant, fully reduced sub-states Ni_a_-SR, Ni_a_-SR′ and Ni_a_-SR′′, in addition to the partially reduced Ni_a_-C state and a small amount of the Ni_a_-L species (Fig. 3a and Table S3†). The same bands, though in different relative ratios, were observed for samples of reduced native MBH (Fig. 3a). The lower intensities of the absorption bands of the H_2_-reduced MBH^C120G^ variant, however, indicate protein instability caused by the C120G exchange, which was not observed for the MBH^C19G^ variant (Fig. 3a).

The EPR spectrum of H_2_-reduced MBH^C120G^ lacks the characteristic Fe–S cluster-derived signal present in reduced native MBH (Fig. 3b). Instead, very weak signals occurred in the g-value range from 2.05 to 1.80, which we attribute to traces of one or more reduced proximal [4Fe–4S] cluster species. EPR measurements at various temperatures and microwave powers indeed revealed different spin relaxation properties (Fig. S11†), suggesting the presence of at least two electronically and structurally different species. This is consistent with the crystallographic data, which suggest multiple conformations of the reduced proximal cluster (Fig. 2c). The corresponding resonance Raman spectra revealed the disappearance of all cluster-related Fe–S modes in H_2_-reduced MBH^C120G^ crystals, indicating the complete reduction of the Fe–S cluster relay (Fig. S10†).

Re-oxidation of H_2_-reduced MBH^C120G^ did not result in the re-appearance of Ni_r_-B-associated EPR signals with the same intensity as initially observed in the oxidized enzyme (Fig. S9a†). In addition, the amount of Ni_ia_-S species (∼45%) was almost as large as that of Ni_r_-B (∼55%) as revealed by IR spectroscopy (Fig. S9b†). These results support the inherent instability of the solubilized MBH^C120G^ variant upon repeated redox treatments.

### H_2_ turnover activity of the MBH variants in the presence of O_2_

**Fig. 4:**
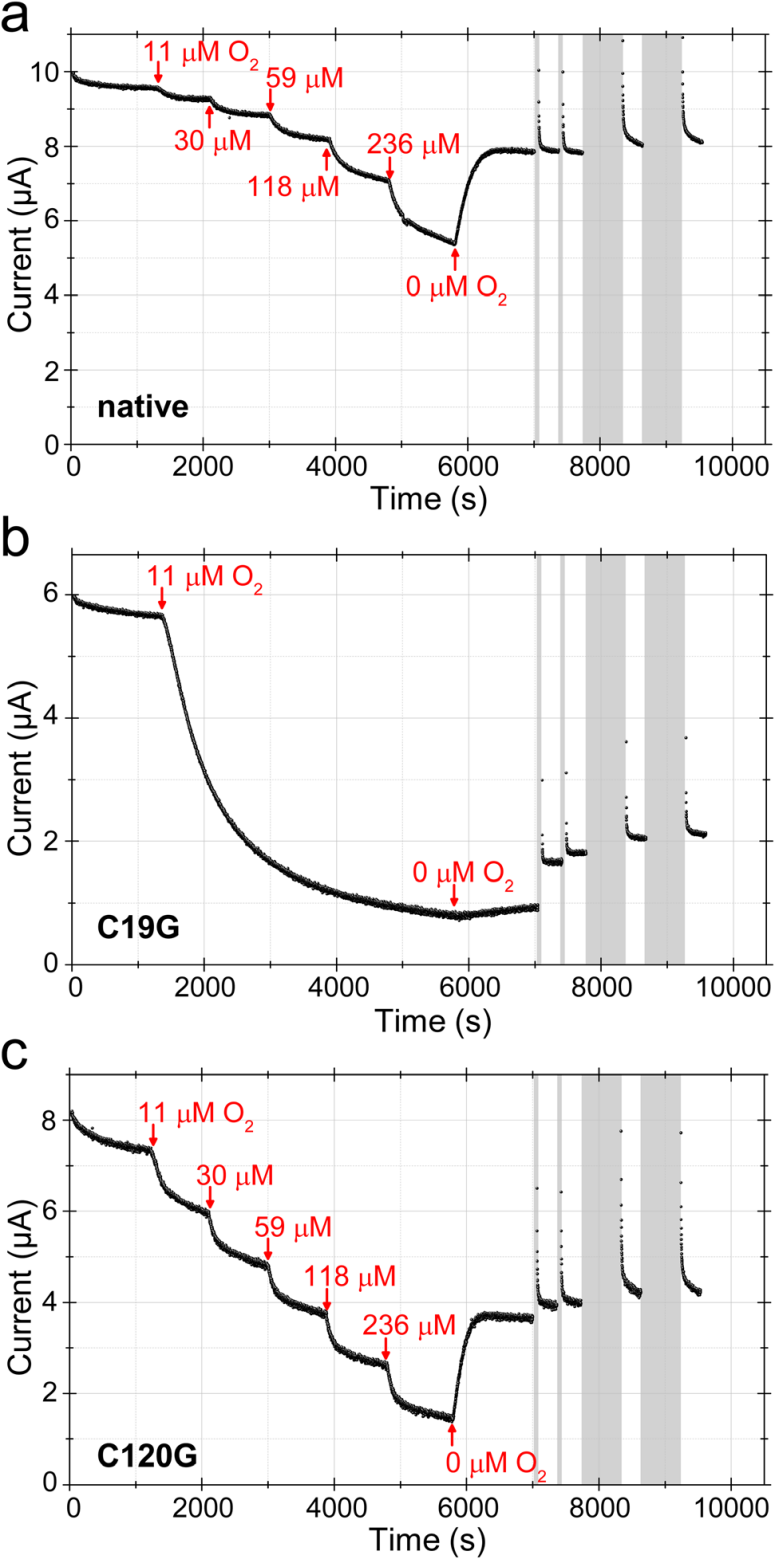
Chronoamperometry to monitor the inhibition of H_2_ oxidation of (a) native MBH, (b) MBH^C19G^, and (c) MBH^C120G^ upon exposure to increasing concentrations of O_2_ in 80% H_2_ using PGE/MWNT/Py-enzyme modified electrodes. Initial current was measured at 0 V in 80% H_2_ (606 μM) for 1200 s. Then, an initial concentration of 1% O_2_ (11 μM) was introduced into the headspace in the case of all proteins. Exposure to 1% O_2_ was allowed to proceed for 900 s for native MBH and MBH^C120G^, and subsequently, the O_2_ concentration was increased stepwise as indicated (corresponding to 1, 2.5, 5, 10 and 20% O_2_). The current continuously dropped even when MBH^C19G^ was exposed to as little as 1% O_2_, so the concentration was not further increased. After exposure to O_2_ for a total of 4600 s for each variant, the headspace was returned to 80% H_2_ in Ar to flush the O_2_ from the cell for 1200 s. A series of low potential (−0.659 V) pulses were then applied (2 for 60 s and 2 for 600 s, grey bars) to provide extra driving force for the re-activation of aerobically-formed species, always returning to 0 V to compare the recovered current to that initially measured. Other conditions: *T* = 30 °C, pH = 5.5, *ω* = 3500 rpm, Ar carrier gas, total gas flow = 500 scc min^−1^.

Protein film electrochemistry (PFE) is an established method to investigate the H_2_/H^+^-cycling properties of (bio-)catalysts immobilized onto conducting surfaces such as pyrolytic graphite electrodes or metal (oxides) with biocompatible coatings.^31–34^ The measured currents are proportional to enzymatic turnover making the technique particularly useful for testing the influence of inhibitory gases such as O_2_ during H_2_ activation. Here, it is important that the gas composition is tightly controlled during the time the catalytic current is monitored, since changes in activity in response to a particular gas may be slow. The electrochemical cell is thus sealed, and equipped to allow a controlled mixture of gases to flow through the headspace above the solution while the electrode is rotated. Gases to be tested are introduced or removed by adjusting a mass flow controller that rapidly mixes the different components in a precise manner (H_2_, Ar, O_2_*etc.*).^35^ PFE has already been employed to investigate the MBH^C19G/C120G^ variant on a bare pyrolytic graphite electrode.^9^ The protein rapidly lost its H_2_-oxidizing activity as soon as O_2_ was added to the assay solution, which was in deep contrast to the observations for native MBH.^9^ Here, we tested the capabilities of the MBH^C19G^ and MBH^C120G^ proteins to sustain H_2_ conversion in the prolonged presence of O_2_. To avoid irreversible ‘loss’ of protein film from the electrode, seen as a continuous loss of current over time,^9^ film stability can be improved by covalently attaching the enzyme onto pyrolytic graphite electrodes coated with pyrene-butyric acid-modified multi-walled carbon nanotubes (MWNT).^36^ The activity of MBH^C19G^ and MBH^C120G^ was followed by cyclic voltammetry (CV) in the presence of 80% H_2_ (606 μM) under otherwise anoxic conditions (Fig. S12†). Both variants showed the typical overpotential of about 80 mV for the onset of catalysis and similar inactivation kinetics under oxidizing conditions at higher potential. However, they reacted differently to short pulses of O_2_ (Fig. S12†), prompting us to investigate the effect of prolonged O_2_ exposure on the H_2_ oxidation activity of the MBH variants in chronoamperometric experiments (Fig. 4). The potential was set to 0 V to avoid direct O_2_ reduction by the electrode. Native MBH showed the typical behavior upon O_2_ treatment.^37,38^ Each O_2_ addition led to a distinct current drop (Fig. 4a). The resulting steady state current derives from active enzyme, the level of which is determined by the rate of O_2_-mediated inactivation and the rate of re-activation. A prerequisite for this behavior is that unready states of the active site are not formed upon O_2_ incubation, which would lead to a constant decline of the current. An important feature of the native MBH is its capability to regain a significant portion of activity as soon as O_2_ is removed from the cell headspace (Fig. 4a).^39^ Stepwise addition of O_2_ to H_2_-oxidizing MBH^C120G^ variant revealed a comparable current progression as observed for native MBH (Fig. 4b). However, at the highest O_2_ concentration, MBH^C120G^ lost almost 80% of its initial activity, while corresponding loss of native MBH was just ∼50%. As with native MBH, flushing O_2_ from the cell resulted in rapid spontaneous recovery of catalytic activity. In contrast to native MBH, however, a further current increase was observed upon application of a series of short (60 s) and extended (600 s) low potential pulses (−0.659 V). Nevertheless, the current increase was rather small compared to the recovery observed after O_2_ removal (Fig. 4b).

The MBH^C19G^ variant showed a more pronounced response toward O_2_ exposure. Addition of just 1% O_2_ (11 μM) resulted in a continuous drop in H_2_ oxidation current (Fig. 4c). After 4600 s of low-level O_2_ exposure, the current reached a stable level corresponding to less than 20% of the initial activity. In contrast to native MBH and MBH^C120G^, the removal of O_2_ from the solution, resulted in only a marginal recovery of the current. A more significant recovery was achieved, however, after the application of low potential pulses (Fig. 4c).

**Fig. 5:**
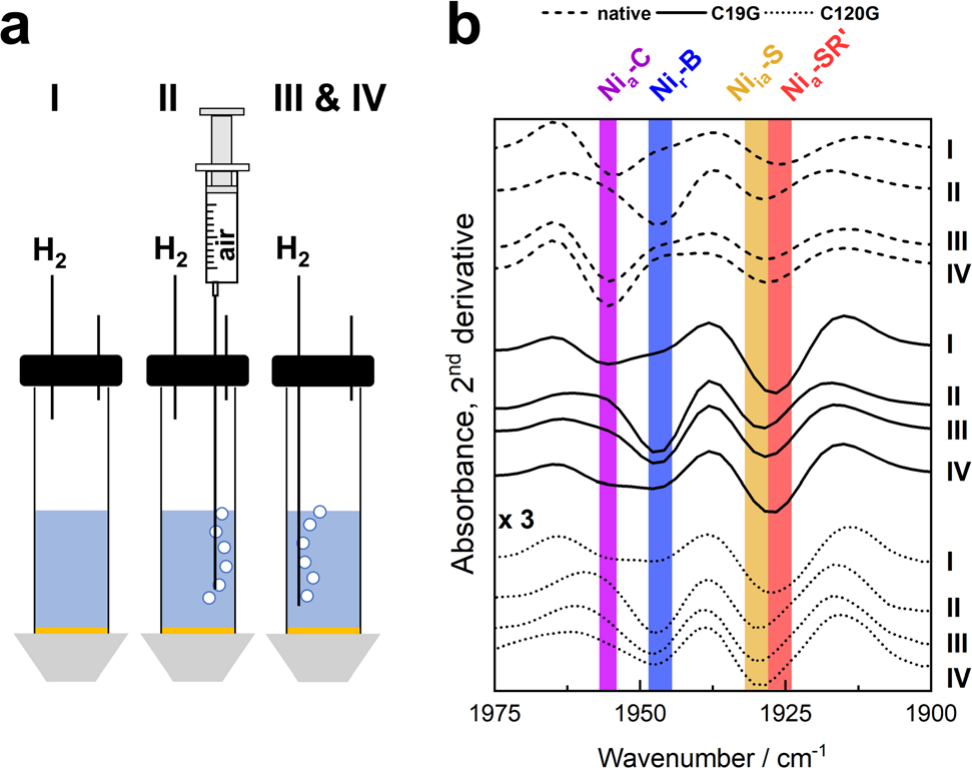
SEIRA spectroscopy of the MBH variants under electrochemical control. Proteins were immobilized on bio-compatibly coated Au electrodes, and spectroscopic changes were followed during chronoamperometry at 0 mV (*vs.* SHE). (a) Experimental set-up and procedure of the experiment. In phase I, the protein solution was subjected to a pure H_2_ atmosphere. Phase II included the stepwise addition of air to the buffer reservoir which was subsequently removed in phase III by flushing with pure H_2_. In phase IV, two low-potential pulses were applied at −400 mV, and the potential was then set back to 0 mV. (b) Second derivatives of the SEIRA spectra in the region characteristic for the CO absorptions recorded at the end of each phase. Spectra of MBH^C120G^ are enlarged by a factor of three (Fig. S14†). The redox states corresponding to specific absorption bands are marked with coloured bars. For further details, see Fig. S13.†

To obtain insight into the nature and distribution of the active site redox states during catalysis, we employed a setup allowing surface-enhanced IR absorption (SEIRA) spectroscopy under electrochemical control (Fig. 5). The MBH variants were immobilized onto a nanostructured Au electrode coated with a self-assembled monolayer, and we carried out a chronoamperometric study by employing similar, but not identical conditions as used for the PFE experiments described above (see Fig. S13† for details). IR spectra were recorded during H_2_ conversion in the absence and presence of O_2_, as well as after flushing out the O_2_ followed by reductive reactivation at negative potentials (Fig. 5 and S13†). Under pure H_2_, the spectra of the native MBH and the variants C19G and C120G were dominated by the catalytic Ni_a_-C and Ni_a_-SR′ states. Furthermore, two inactive states (Ni_r_-B and Ni_ia_-S) were observed in all variants. The amount of the Ni_ia_-S state (CO peak at 1930 cm^−1^) seemed to increase continuously during the chronoamperometric measurements, irrespectively of the gas composition. As the quantity of immobilized hydrogenase did not change during the course of the spectro-electrochemical measurements (Fig. S14†), we interpret the intrinsic decline of the catalytic current (often stated as film loss) with the accumulation of the apparently irreversibly inactive Ni_ia_-S state. The signal attributed to the Ni_r_-B state (CO peak at 1948 cm^−1^), which accumulates in the presence of the H_2_/O_2_ mixture, disappeared completely in native MBH after removal of oxygen. In contrast, the same treatment did not result in the complete elimination of the Ni_r_-B signal for the MBH^C120G^ variant and especially for the MBH^C19G^ variant (Fig. 5b and S13†). Only the application of low-potential pulses under anaerobic conditions resulted in lowering of the Ni_r_-B signals in favor of the population of signals resulting from reduced states. This result is consistent with the level of current recovery during chronoamperometry, which was highest for the MBH^C19G^ variant (Fig. 4c and S13†). Notably, IR bands of other inactive species such as Ni_u_-A were not found in detectable amounts in any of the immobilized MBH variants.

## Discussion

### Substitution of auxiliary cysteines changes iron–sulfur cluster types with unprecedented structural features

Exchanges of the supernumerary cysteines of the proximal [4Fe–3S] cluster had distinct consequences regarding the yield and catalytic activity of all MBH variants. In fact, the single C120G exchange caused the most drastic effect, as both the protein yield and the H_2_-mediated methylene blue reduction capacity (under anoxic conditions) were only about 10% of the corresponding values for the native protein. Despite the low yield, we were able to solve the crystal structures of the MBH^C120G^ variant of the oxidized and H_2_-reduced forms. Among the MBH variants, the MBH^C120G^ protein showed the highest structural plasticity with at least two different states of the proximal Fe–S cluster for each of the two redox conditions. In approximately 50% of the H_2_-reduced MBH^C120G^ crystals, this new cluster resembles the structure of the reduced native [4Fe–3S] cluster, except that the Cys120 thiolate is partially replaced by an exogenous sulfur species (S4) that coordinates Fe3, resulting in a [4Fe–4S] cluster in a non-cubic form. The other 50% does not contain the S4, making Fe3 part of a cubane, one corner of which is occupied by the Cys19-derived sulfur. To our knowledge, such a novel cubic cluster in which one of the inorganic sulfides is replaced by a cysteine sulfur remains unprecedented. The replacement of Cys120 results in a double conformation of Fe3 that correlates with the exo-sulfur species in terms of the occupancy. The proximal Cys_5_[4Fe–4S] cluster in oxidized MBH^C120G^ is even more flexible, forming a new cluster scaffold with altered redox properties and electronic structure. In one fraction of the protein, Fe4 is bound to the backbone nitrogen of Cys20 (Fe4′ position), as observed for the native super-oxidized [4Fe–3S] cluster. The other fraction (Fe4) remains in the equivalent position to that found for the reduced state (Fig. 2c). The observations clearly indicate that Cys19 is the crucial ligand that allows the movement of Fe4 to move in the native proximal [4Fe–3S] cluster of MBH. However, the overall coordination of Fe4 in MBH^C120G^ differs from that in the native [4Fe–3S] cluster. In conclusion, our results underline the stabilizing role of Cys120 in the plasticity and redox function of the Cys_6_[4Fe–3S] cluster. In addition to a change in redox properties, removal of Cys120 resulted in an inappropriate flexibility of the proximal cluster in MBH^C120G^, which, in turn, caused decreased protein stability, as manifested by low protein yield and low abundance of this MBH variant in *C. necator* cells.^9^

The crystal structures of H_2_-reduced MBH^C19G^ and MBH^C19G/C120G^ revealed almost cubic [4Fe–4S] clusters coordinated by the four canonical cysteine residues, which had been expected in case of the C19G/C120G exchange, but experimental evidence was lacking.^9,40^ In MBH^C19G^, however, the presence of Cys120 and the new sulfide (S4) resulted in a certain redox-dependent flexibility of Fe3, which is comparable but not identical to the situation in MBH^C120G^. In H_2_-reduced MBH^C19G^ crystals, a major fraction of Fe3 is not covalently bound to Sγ-Cys120, which is in contrast to the reduced [4Fe–3S] cluster of native MBH. This result can be explained by the fact that sulfide (S4) is a more tightly bound ligand than a thiolate, which leads to coordinative bonding to the shifted Fe3′. The corresponding cubic conformation increases the stability of the cluster. As expected, the Fe4 shift does not occur in the oxidized [4Fe–4S] cluster of MBH^C19G^. Here, most of Fe3 is covalently bound to Cys120 and not to S4, which is similar to the cluster structure of the C19G variant of *E. coli* Hyd-1 resulting in a less negative reduction potential.^41^ Overall, our data show that the covalent bond between Cys120 and Fe3 is crucial for stabilizing the super-oxidized state and thus for the physiological function of the proximal cluster.

Even in the MBH^C19G/C120G^ protein, which contains a four Cys-coordinated [4Fe–4S] species, oxidation resulted in cluster flexibility, indicating that the binding pocket is optimized for an Fe–S cluster of larger dimensions, *i.e.*, the Cys_6_[4Fe–3S] cluster (Fig. 2). Thus, the Cys_4_[4Fe–4S] cluster in MBH^C19G/C120G^ is similar but not identical to the classical Cys_4_[4Fe–4S] cluster of O_2_-sensitive [NiFe]-hydrogenases.

### Insight into the maturation of the proximal Cys_6_[4Fe–3S] cluster

**Fig. 6:**
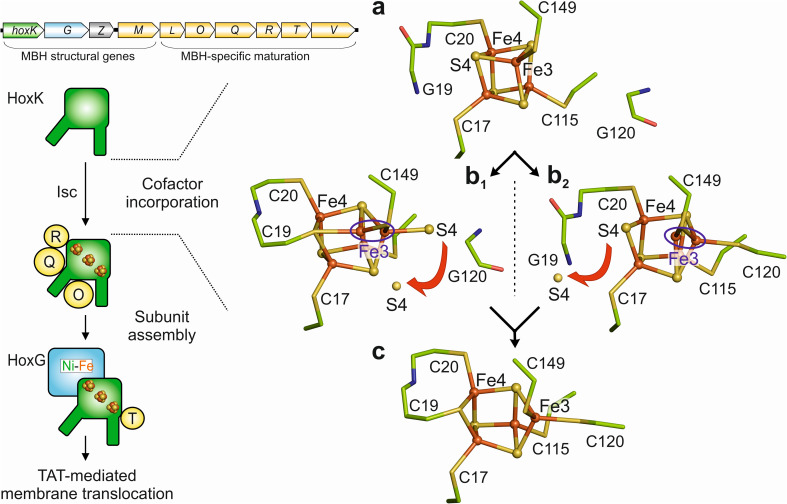
Possible maturation pathway(s) of the proximal [4Fe–3S] cluster. (Left) Schematic overview of the MBH maturation, which requires dedicated maturases (yellow circles) encoded in the MBH gene cluster (top).^42^ (Right) (a) The first step would be the insertion of a standard cubane [4Fe–4S] cluster, followed by either (b_1_) covalent binding of Cys19 to Fe4 and Fe1 or (b_2_) binding of Cys120 to Fe3. Binding of the first additional cysteine is expected to lead to a destabilization of the cubane and subsequent release of S4. This would allow covalent binding of the second additional cysteine, leading to stabilization of the classical [4Fe–3S] cluster conformation (c). The proximal cluster, selected amino acids and water molecules are depicted as ball/sticks, sticks and red spheres respectively. The destabilization at Fe3 is highlighted by a blue circle. Red arrows indicate the release of S4.

The different crystal structures of the MBH variants prompted us to propose a possible scheme of the transformation process from a [4Fe–4S] cluster to a [4Fe–3S] species (Fig. 6). The first step is presumably the IscU-assisted insertion of a preformed [4Fe–4S] cluster,^42^ which becomes attached to the canonical cysteines Cys17, 20, 115 and 149 (also present in all periplasmic, membrane-associated [NiFe]-hydrogenases carrying a [4Fe–4S] cluster in the proximal position) of the MBH small subunit. The [4Fe–4S] cluster as present in the H_2_-reduced MBH^C19G/C120G^ may represent this maturation intermediate, as this species is the most stable among the cluster variants. However, this hypothesis may be supported by future studies on MBH variants in which the canonical cysteine residues were exchanged. We also hypothesize that cluster rearrangement is subsequently triggered by one of the two additional cysteines, Cys19 or Cys120. In the first scenario, Cys19 would initiate the rearrangement, and its thiolate takes over the coordination of Fe1 and Fe4, replacing sulfide S4. The released S4 could either be delivered to the solvent (assuming that the binding pocket of the proximal cluster is exposed to the solvent during maturation) or swings over towards the position of the thiol group of Cys120. Either of these steps would be accompanied by a shift of Fe3 from the corner position of the cubane. These transformation steps would result in the Cys_5_[4Fe–4S] cluster as present in the reduced MBH^C120G^ variant. In the second scenario, the Cys120 would first covalently bind Fe3, which should lead to a non-tetrahedral coordination of S4 (represented by the oxidized Cys_5_[4Fe–4S] cluster of MBH^C19G^) and its subsequent release. Both scenarios would eventually lead to covalent binding of Cys19 and Cys120 to the corresponding iron ions of the mature Cys_6_[4Fe–3S] cluster (Fig. 6). Under aerobic conditions, this transformation process appears to require specific auxiliary and protective proteins^42,43^ because the proximal Fe–S cluster in the isolated small subunit is solvent-exposed and therefore susceptible to O_2_.^6^ Interestingly, the EPR spectrum of the oxidized C19G variant of MBH (Fig. 3b) shows striking similarities to that of MBH isolated from a *C. necator* mutant strain in which the rubredoxin-like maturase-encoding gene *hoxR* was deleted.^44^ This is the first indication that *HoxR* enables the correct bridging coordination of Cys19 to Fe1 and Fe4 under aerobic conditions for both of the proposed maturation pathways.

### Cysteine exchanges restrict the number of redox transitions mediated by the proximal cluster

IR spectroscopy revealed that all three oxidized MBH derivatives investigated in this study form predominantly the Ni_r_-B state, which is consistent with the crystal structure and EPR data. The sulfoxygenated cysteine observed in oxidized MBH^C19G^ crystals is not reflected by the spectroscopic data, probably because of its low occupancy, which is even lower in H_2_-reduced crystals, indicating that this modification is slowly reactivatable rather than irreversibly inactive. In addition to MBH^C19G^ and MBH^C120G^, the oxidized MBH^C19G/C120G^ protein^9^ forms preferentially the Ni_r_-B state, showing that the manifold engineered proximal clusters does not necessarily lead to accumulation of Ni_u_-A.

Concerning the EPR analysis, the simplest EPR spectrum was observed for oxidized MBH^C120G^. It consists of an axial signal attributed to the medial [3Fe–4S]^1+^ cluster in addition to the typical rhombic Ni_r_-B signal (Ni^3+^). In contrast to the corresponding spectrum of the native MBH, the proximal cluster of the oxidized MBH^C120G^ remains diamagnetic, as no additional signals and no splitting or broadening of the signals for Ni_r_-B and the medial cluster were observed. The same observation has been made for the oxidized C19G/C120G exchange variants of MBH and *E. coli* Hyd-1.^9,30^ In addition, H_2_-reduced MBH^C19G/C120G^ and Hyd-1^C19G/C120G^, were EPR-silent, suggesting a fully reduced active site and a diamagnetic medial [3Fe–4Fe]^0^ cluster. This observation, in turn, is consistent with the EPR spectrum of reduced MBH^C120G^, which contained only very weak signals interpretable as reduced Fe–S cluster species. The spectrum is also consistent with the predominant formation of EPR-silent, fully reduced Ni_a_-SR sub-states as observed by IR. Although MBH^C120G^, MBH^C19G/C120G^, and Hyd-1^C19G/C120G^ showed catalytic activity, their proximal cluster remains non-detectable by EPR after reduction, possibly caused by the presence of an integer spin or an unusual non-integer high spin configuration that is not detectable under the applied measurement conditions. We conclude that the Cys_5_[4Fe–4S] cluster of MBH^C120G^ and most likely also the proximal Cys_4_[4Fe–4S] clusters of MBH^C19G/C120G^ and Hyd-1^C19G/C120G^ perform only one low-potential electron transition.^15,17,45^ This is in accordance with our protein tunneling calculations of the corresponding crystal structures that the exchange of Cys120 with a smaller Gly residue increases the solvent accessibility of the proximal Fe–S cluster in MBH^C120G^ and MBH^C19G/C120G^ crystals (Fig. S6†). This shifted solvent accessibility could contribute to confer a single low redox potential transition.^46^

In contrast, a major fraction of the Cys_5_[4Fe–4S] cluster of the MBH^C19G^ variant was EPR-active under oxidizing conditions, whereas no Fe–S cluster-related EPR signal was observed under reducing conditions. Thus, the proximal Cys_5_[4Fe–4S] cluster of the MBH^C19G^ variant mediates a 3+ to 2+ transition at higher potentials, while the 2+ to 1+ transition does not take place within a physiological potential range.^46^ This conclusion is consistent with data obtained for Hyd-1^C19G^,^41^ for which a redox titration in combination with EPR spectroscopy and computational studies revealed that the proximal [4Fe–4S] cluster is capable of undertaking the “high-potential” transition of the two redox transitions mediated by the native [4Fe–3S] site.^41^ The associated redox potential of +240 mV was very close to that of the super-oxidized/oxidized couple of the [4Fe–3S] cluster (230 mV).^30^ This result agrees with our observations for the proximal cluster of the MBH^C19G^ variant, which also seems to allow only the high-potential redox transition.

### Accumulation of inactive states during H_2_ cycling under oxygenic conditions

According to both the cyclic voltammetry and the chronoamperometric PFE experiments, all MBH variants lost a certain amount of activity upon O_2_ incubation, with native MBH being least and MBH^C19G^ most affected. In the case of MBH^C19G^ and partially also for MBH^C120G^, O_2_ exposure led to the accumulation of inactive enzyme species that could in part be reactivated by applying a very negative electrode potential. Our PFE results are in line with those obtained for *E. coli* Hyd-1 variants carrying equivalent exchanges of the supernumerary cysteines.^40^ Accompanying spectro-electrochemical SEIRA studies indicate that the current decrease upon O_2_ incubation is related to the accumulation of two inactive states of the catalytic center in all MBH variants. The first one is the inactive Ni_ia_-S state, the amount of which increased steadily during the experiment, regardless of which gas mixture was present, indicating no direct correlation between Ni_ia_-S state accumulation and O_2_-induced inactivation. Besides Ni_ia_-S (and the reduced states), all three investigated MBH variants formed the Ni_r_-B state during H_2_/H^+^-cycling in the presence of O_2_. The fact that for the MBH^C19G^ protein in the Ni_r_-B state, current recovery occurred mainly after applying negative potential pulses indicates major kinetic limitations regarding its reactivation ability, which resulted in only a marginal current recovery upon O_2_ removal. In fact, the redox potential of the Ni_r_-B state of the *C. necator* MBH lies at −110 mV.^47^ Reactivation of the Ni_r_-B state requires one proton and one electron (Fig. 1), the latter of which must be supplied in a reverse fashion by the iron–sulfur cluster chain of the MBH small subunit. With the medial [3Fe–4S] cluster and the proximal Cys_5_[4Fe–4S] cluster, the MBH^C19G^ protein carries two rather high-potential clusters in a row, which creates a more severe kinetic barrier for the efficient reactivation of Ni_r_-B.

## Conclusions

### Re-interpretation of the oxygen tolerance mechanism conferred by the [4Fe–3S] cluster

The oxidized active site of all MBH derivatives seems to reside predominantly in the Ni_r_-B state, which we consider as a very important observation. The bridging hydroxy ligand prevents the active site from interacting with other substrates. Our results indicate that generation of the Ni_r_-B state in oxygen-tolerant MBH is not intimately linked to the two-electron transfer capacity of the unique [4Fe–3S] cluster, which is actually impeded in all proximal cluster variants of the MBH. This proposal is supported by previous results from ascorbate-treated, partially reduced native MBH, which resulted in the formation of a mixture of super-oxidized and oxidized forms of the [4Fe–3S] cluster, while the catalytic center remained in the Ni_r_-B state.^17^ Similarly, the oxidized crystal structures of Hyd-1 from *E. coli* and MBH of *H. marinus* contained an active site fully occupied with a hydroxide ligand, in combination with mixtures of oxidized/reduced and super-oxidized [4Fe–3S] clusters. Only treatment with the strong oxidant ferricyanide resulted in exclusive binding of Fe4 to the backbone nitrogen of Cys20.^13,15^ These observations suggest that formation of the super-oxidized [4Fe–3S] cluster is mainly triggered by the redox status of the iron–sulfur relay, which interacts with the quinone pool of the respiratory chain in the native environment.^29^ This assumption implies that *in vivo* only a completely oxidized iron–sulfur cluster chain (*i.e.* lacking any reducing capacity as might occur during H_2_ oxidation under aerobic conditions) leads to the formation of the super-oxidized state of the [4Fe–3S] cluster, as suggested previously^45^ and supported by the fact that O_2_-mediated full oxidation to the super-oxidized state exclusively takes place if the MBH is still coupled to the cellular quinone pool (Fig. S9†).

Removal of the active site OH ligand under H_2_/electron-limiting conditions, regenerating the Ni_a_-S state, leaves the active site unprotected and vulnerable to O_2_-mediated inactivation. Reduction of the [4Fe–3S] cluster by one electron (originating from the Fe–S cluster chain), however, results in the movement of Fe4 back into the Fe–S cluster core. As this reorganization is time-consuming,^45^ the [4Fe–3S]^5+^ cluster functions as an implemented time-delay preventing premature reactivation of the Ni_r_-B state in the case of H_2_ scarcity under otherwise oxic conditions. Importantly, the active site protection mechanism described above does not preclude the previously described model of O_2_ detoxification by four electrons and four protons delivered to the active site in the reverse direction to reduce O_2_ to harmless water.^9,14,18,19,37,48^ In the proposed new mechanism, activity can be switched off under electron-deficient conditions even before O_2_ would gain access to the catalytic center. In fact, *Knallgas* bacteria such as *C. necator* usually face an (micro)aerobic environment in which H_2_ occurs transiently and only in traces.^4^

## Experimental section

### Material and methods

#### Protein expression and purification

##### Construction of MBH^C19G^ and MBH^C120G^

The amino acid exchanges C19G and C120G in the small MBH subunit HoxK were introduced by the QuikChange (Agilent) method using primers #793/#794 for C19G and #795/#796 for C120G^9^ on plasmid pCH1229.^37^ The modified plasmids were digested with SpeI/SmaI and the resulting 1171 bp fragments were transferred to SpeI/SmaI-cut pJH2430 (Litmus28 containing P_MBH_-*hoxK*_Strep_-*hoxV*) resulting in plasmids pJH5116 (C19G) and pJH5127 (C120G). Plasmids pJH5116 and pJH5127 were digested with SpeI/BstZ17I and the resulting 2581 bp fragments were ligated into SpeI/BstZ17I-cut pCH785 (ref. 49) resulting in pJH5143 (C19G) and pJH5148 (C120G). These plasmids were digested with SpeI/XbaI and the resulting 21.5 kbp fragments were ligated into XbaI-cut pEDY309 (ref. 50) resulting in pJH5175 (C19G) and pJH5179 (C120G). pJH5175 and pJH5179 were conjugated using *E. coli* S17-1 (ref. 51) into *C. necator* HF210 resulting in strains HP83 (C19G) and HP84 (C120G).

##### Construction of MBH^C19G/C120G^

The double amino acid exchange C19G/C120G in the small MBH subunit HoxK was introduced by digesting pCH1487 ^9^ with SpeI/BstZ17I and ligating the resulting 2581 bp fragment into SpeI/BstZ17I-cut pCH785 resulting in plasmid pJH4052. The latter was digested with SpeI/XbaI and the resulting 21.5 kbp fragment was ligated into XbaI-cut pEDY309 resulting in plasmid pJH4081 which was conjugated into *C. necator* HF210 resulting in strain HP29. Recombinant *C. necator* strains were cultivated and the MBH variants purified as described elsewhere.^51^

#### Crystallization, X-ray data collection and structure analysis

The purified MBH^C19G^, MBH^C120G^ and MBH^C19G/C120G^ variants were crystallized under either aerobic (oxidizing) or anaerobic, reducing conditions (in the presence of H_2_) as described previously.^14,17^ In brief, the individual MBH variants were crystallized by applying the sitting-drop vapor diffusion method in 24-well Linbro plates (Jena Bioscience) at a concentration of 10 mg mL^−1^ at 277 K and 283 K for oxidized and reduced crystals, respectively. The reservoir solution contained 20–30% polyethylene glycol 3350, 100 mM bis-(2-hydroxy-ethyl)-amino-tris(hydroxymethyl)-methane buffer at pH 5.5–6.5. The drop ratio of precipitant and protein solution varied between 0.5 and 2. Brownish crystals appeared within 2–4 days and upon collection were directly flash-frozen in liquid nitrogen (LN2).

Diffraction data were collected at the synchrotron facilities BESSY II (Berlin, Germany) and ESRF (Grenoble, France). The best diffracting data for H_2_-reduced MBH^C19G/C120G^ was collected at BESSY II, beamline BL14.1 (ref. 52) with a Rayonix MX-225 detector at a wavelength of 0.918 Å (PDB entry 8POV). For oxidized MBH^C19G/C120G^, data collection was executed at ESRF, beamline ID29 (ref. 53) with a PILATUS M6-F detector at a wavelength of 0.976 Å (PDB entry: 8POU). For anomalous data collection, the wavelength was shifted towards 1.734 Å. The best diffracting data for oxidized and H_2_-reduced MBH^C19G/C120G^ were collected at ESRF, beamline ID29 (ref. 53) with a PILATUS M6-F detector at a wavelength 0.969 Å and 1.00 Å, respectively (PDB entries 8POW and 8POX). Data for oxidized and H_2_-reduced MBH^C120G^ crystals were collected at the ESRF, beamline ID29 (ref. 53) with a PILATUS M6-F detector at a wavelength of 0.969 Å (PDB entries: 8POY and 8POZ). Additionally, anomalous data at 1.734 Å was recorded for MBH^C120G^. Images were indexed, integrated and scaled using the *XDS* program package^54^ and the *CCP4* program *SCALA*.^55,56^ All crystals showed the orthorhombic space group *P*2_1_2_1_2_1_. Table S2† summarizes the statistics for crystallographic data collection and structural refinement. Initial phases for the MBH structures were obtained by molecular replacement using crystal structures of H_2_-reduced native (reduced) MBH (PDB entry 3RGW^14^) and as-isolated (super-oxidized) native MBH (PDB entry 4IUB^17^) as the initial search models for the MBH variants. Molecular replacement was achieved using the *CCP4* program *PHASER*.^57^ Phasing and heavy atom search for analyzing anomalous data were performed by using the *SHELX* package.^58^ Statistics for anomalous crystallographic data collection are provided in Table S2.† The structures of the MBH variants were refined with the programs *PHENIX* and *CCP4* program *REFMAC5* using refinement strategies (*inter alia* real-space refinement, *B*-factor refinements) and simulated-annealing (slow cooling protocol, maximum likelihood target function, energy minimization) to overcome potential phase biases.^55,59–61^ First water molecules were depicted with the automatic search method in *PHENIX*.^60^ Manual model building and electron density interpretation were performed in *COOT*.^62^ After every round of manual model building, energy minimization and *B*-factor refinement were performed with *REFMAC5*.^61,63^ The final crystal structures were validated using the *RCSB PDB* Validation Server,^64^*MolProbity* server^65^ and *WHAT IF* server.^66^ Solvent accessibility was calculated in *PyMOL* using the program *CAVER3.0*.^67^ All molecular graphics representations were created in *PyMOL*.^68^

#### Protein film electrochemistry

To prepare each film of non-covalently bound enzyme on pyrolytic graphite ‘edge’ (PGE) disk electrodes (geometric area 0.03 cm^2^), the electrode was first polished for 30 s with an aqueous slurry of α-alumina (1 μm, Buehler) and sonicated for 5 s in purified water, before MBH variant solution (1.5 μL, 0.2–1 μg of enzyme, pH 5.5) was repeatedly applied and withdrawn from the electrode surface over a period of 10–30 s. The electrode was then rinsed in purified water and used immediately.

Covalent immobilization of MBH on electrodes was performed as described previously.^69^ In each case, a dispersion of multi-walled carbon nanotubes (MWNT) in DMF (16 μL of 1 mg mL^−1^) was placed on the surface of a PGE-disk electrode and allowed to dry overnight. The MWNT-coated PGE electrodes (geometric area 0.2–0.28 cm^2^) were then coated with a solution of 1-pyrenebutyric acid (Py) in DMF (10 mM) for 1 h to obtain strong π–π stacked MWNT-Py assemblies on PGE (PGE/MWNT/Py). The electrodes were rinsed in purified water and the free carboxylic groups of Py were activated for 20–40 min using a freshly prepared solution of 1-ethyl-3-(3-dimethylaminopropyl) carbodiimide (EDC, 0.4 M) + *N*-hydroxy sulfosuccinimide (NHSS, 0.1 M) before treating with MBH variant solutions at room temperature for 60 min to make covalent attachments *via* the surface lysine residues (∼33 for MBH from *C. necator*). This procedure resulted in modified electrodes denoted PGE/MWNT/Py-MBH. The electrodes were then rinsed in purified water and used immediately or stored at room temperature in 50 mM potassium phosphate, 100 mM NaCl buffer, pH 5.5.

Protein film electrochemistry experiments were carried out using an electrochemical analyzer (Autolab PGSTAT128N) controlled by a PC operating NOVA software (EcoChemie) in an anaerobic glovebox (M. Braun) comprising a N_2_ atmosphere (O_2_ <2 ppm). The enzyme-coated electrodes were placed in enzyme-free buffered electrolyte (50 mM potassium phosphate, 100 mM NaCl, pH 5.5) so that all enzyme molecules addressed in the experiments were subjected to the same regime of strict potential control. In all experiments the electrode was rotated at a constant rate (>2500 rpm) to provide efficient supply of substrate and removal of product. The electrode rotator (EcoChemie) was placed in a specially designed, gas-tight, glass electrochemical cell incorporating a Pt wire counter electrode. A saturated calomel reference electrode (SCE) was contained in a sidearm containing 100 mM NaCl, separated from the main cell compartment by a Luggin capillary. Potentials (*E*) are quoted with respect to the standard hydrogen electrode (SHE) using the conversion *E*_SHE_ = *E*_SCE_ + 0.241 V at 25 °C.^70^

Experiments were performed in phosphate buffer (50 mM; Fisher Scientific) containing NaCl (100 mM; Analytical Grade, Fisher Scientific) and titrated to the desired pH at the experimental temperature. Experiments were performed under gas mixtures of H_2_ (Premier Grade, Air Products), Ar, and O_2_ (BOC gases) created using mass flow controllers (Sierra Instruments).

#### Sample preparation for IR and EPR measurements

For measurement of oxidized MBH variants, as-purified protein samples were concentrated with an Amicon Ultra concentrator to approx. 0.2 mM. For reduction, as-purified MBH samples were transferred into an anaerobic glovebox containing a forming gas atmosphere (5% (v/v) H_2_, 95% (v/v) N_2_) and incubated under a stream of humidified H_2_ for 45 min. IR transmission cells and EPR tubes were purged with H_2_ prior to protein transfer to avoid re-oxidation by residual oxygen. For re-oxidation, reduced samples were exposed for 60 min to a stream of humidified air. EPR samples were transferred into quartz EPR tubes, flash-frozen in cold ethanol (210 K) and stored in liquid N_2_ (77 K) for further analysis.

#### IR spectroscopy

All IR transmission spectra were measured at 10 °C in a gastight cell with a sandwich configuration (optical path length 50 μm) using a Bruker Tensor 27 FTIR spectrometer equipped with a liquid nitrogen cooled MCT detector and controlled by the Bruker OPUS 7.0 software. The sample compartment was purged with dry air. Absorbance spectra of the sample were calculated using single-channel spectra of the pure buffer as reference.

#### EPR spectroscopy

All EPR measurements were carried out on a Bruker EMXplus spectrometer equipped with an ER 4122 SHQE resonator, an Oxford EPR 900 helium flow cryostat and an Oxford ITC4 temperature controller. Baseline correction was carried out by subtracting a buffer spectrum measured under the same conditions. Spectra were further corrected by using a polynomic or spline function based on broad spectral regions where no signal occurs. Experimental parameters: microwave power: 1 mW; microwave frequency: 9.29 GHz; modulation amplitude: 10 G; modulation frequency: 100 kHz.

#### RR spectroscopy

Resonance Raman spectra were measured on a LabRam HR-800 Jobin Yvon confocal Raman spectrometer connected to a charge-coupled device (CCD) cooled with liquid-N_2_. Experiments were performed at 80 K using a liquid-N_2_-cooled cryo-stage (Linkam Scientific instruments) for temperature control. The Ar^+^ ion laser with an excitation wavelength of 458 nm was focused at a 2–4 μm spot on the surface of the single crystal with a beam power of 1–2 mW. For frequency calibration of the spectra, toluene was used as an external standard.

#### SEIRA spectro-electrochemistry

SEIRA spectroscopic measurements were performed in the Kretschmann-ATR configuration using a Si prism under an angle of incidence of 60°. On the flat surface of the prism thin, nanostructured Au films were formed by electroless (chemical) deposition. The corresponding SEIRA spectra were recorded from 4000 to 1000 cm^−1^ with a spectral resolution of 4 cm^−1^ on a Bruker IFS 66 v/S spectrometer equipped with a photovoltaic MCT detector. For a single spectrum, 400 scans were added up.^71^ For the protein immobilization, biocompatible surfaces were created by immersing Au electrodes for 12 h in an ethanolic solution of 6-mercaptohexanoic acid and 6-mercapto-1-hexanol in a 1 : 9 ratio, with initial concentrations of 1 mM. Thereby, a mixed self-assembled monolayer (SAM) was formed on the electrode surface. Subsequently, the MBH variants were immobilized according to ref. 33 and 71. The spectro-electrochemical measurements were performed using a three-electrode setup that employed the electroless-deposited gold film as a working electrode (geometric area of 0.79 cm^2^), a Pt mesh as a counter electrode, and an Ag/AgCl 3 M KCl (Dri-Ref, WPI) reference electrode. A schematic representation of the homemade SEIRA spectro-electrochemical cell used in this work is depicted elsewhere.^72^ Electrochemical control was achieved using a Metrohm μAutolab potentiostat.

## Data availability

All experimental data and crystallographic analyses are available in the ESI† file and the raw data are available from the authors.

## Author contributions

P. S., O. L and I. Z. conceived and supervised the project. J. F. and O. L. constructed the MBH variants. S. F. and J. F. purified the MBH samples and analyzed them biochemically. A. S., J. K., S. F., J. F., and P. S. managed the MBH crystallization. A. S., J. K., and P. S. collected the X-ray and anomalous diffraction data, performed data processing and solved the MBH structures. A. S., J. K. and P. S. refined and interpreted the MBH structures. C. L. recorded and analyzed EPR data supported by C. T. IR and RR and spectro-electrochemistry data were obtained and analyzed by S. K., C. L. and I. Z. R. M. E. and F. A. A. recorded and analyzed electrochemistry data; A. S., J. K., S. F., C. L., S. K., I. Z., O. L. and P. S. wrote the paper with contributions from all co-authors.

## Conflicts of interest

The authors declare no competing financial interests. Correspondence and requests for materials should be addressed to P. S. (patrick.scheerer@charite.de), O. L. (oliver.lenz@tu-berlin.de), and I. Z. (ingo.zebger@tu-berlin.de).

## Supplementary Material

SC-014-D3SC03739H-s001
