# Supplementary material for: Stepwise conversion of the Cys6[4Fe–3S] to a Cys4[4Fe–4S] cluster and its impact on the oxygen tolerance of [NiFe]-hydrogenase
Source: Chem Sci. 2023 Sep 20;14(40):11105–20. doi: 10.1039/d3sc03739h (PMC10583674; doi:10.1039/d3sc03739h)
Supplement: SC-014-D3SC03739H-s001 [file SC-014-D3SC03739H-s001.pdf]

Electronic Supplementary Material (ESI) for Chemical Science.
This journal is © The Royal Society of Chemistry 2023

Supporting Information

Stepwise conversion of the Cys_6[4Fe-3S] to a Cys_4[4Fe-4S] cluster and its impact on oxygen tolerance of [NiFe]-hydrogenases

Andrea Schmidt[a‡], Jacqueline Kalmsa[‡§], Christian Lorent[b‡], Sagie Katz[b‡], Stefan Frielingsdorf[b‡], Rhiannon M. Evans[c], JohannesFritsch[b¶], Elisabeth Siebert[b], Christian Teutloff[d], Fraser A. Armstrong[c], Ingo Zebger[b*], Oliver Lenz[b*], Patrick Scheerer[a*]

[a] Charité – Universitätsmedizin Berlin, corporate member of Freie Universität Berlin and Humboldt-Universität zu Berlin, Institute of Medical Physics and Biophysics (CC2), Structural Biology of Cellular Signaling, Charitéplatz 1, 10117 Berlin, Germany.

[b] Institut für Chemie, Biophysical Chemistry, Technische Universität Berlin, Straße des 17. Juni 135, 10623 Berlin, Germany.

[c] Department of Chemistry, University of Oxford, OX1 3QR Oxford, United Kingdom.

[d] Department of Physics, Freie Universität Berlin, Arnimallee 14, 14195 Berlin, Germany.

[‡] These authors contributed equally to this work.

[§] *Current address:* UCB Pharmaceutical Manufacturing, 208 Bath Road, Slough, SL1 3WE, UK.

[¶] *Current address:* Leopoldina, Nationale Akademie der Wissenschaften, c/o ABC Business Center, Friedrichstraße 79, 10117 Berlin, Germany.

* Correspondence should be addressed to P.S. (patrick.scheerer@charite.de), O.L. (oliver.lenz@tu-berlin.de), and I.Z. (ingo.zebger@tu-berlin.de)

Table of content

Figure S1. Proximal Fe-S clusters of oxygen-tolerant and oxygen-sensitive [NiFe]-hydrogenases. page S3
Figure S2. Proximal Cys_5[4Fe-4S] cluster structures in reduced and oxidized MBH^{C19G}. page S4
Figure S3. Proximal Cys_5[4Fe-4S] cluster structures in reduced and oxidized MBH^{C120G}. page S5
Figure S4. Proximal Cys_4[4Fe-4S] cluster structures in reduced and oxidized $MBH^{C19G/C120G}$. page S6
Figure S5. Comparison of the proximal Fe-S clusters of native MBH, $MBH^{C19G/C120G}$, and O_2-sensitive [NiFe]-hydrogenase. page S7
Figure S6. Solvent accessibility of the proximal Fe-S cluster in the MBH variants. page S8
Figure S7. [NiFe]-active site structures of reduced and oxidized MBH variants. page S9
Figure S8. EPR spectra of oxidized native MBH, MBH^{C19G}, and MBH^{C120G} measured at 80 K. page S10
Figure S9. EPR and IR spectra of re-oxidized samples of native MBH, MBHC19G and MBHC120G. page S11
Figure S10. Resonance Raman (RR) spectra of single crystals of oxidized and reduced native MBH, MBH^{C19G} and MBH^{C120G}. page S13
Figure S11. Temperature and microwave power dependence of the EPR signals of H_2-reduced MBH^{C120G}. page S14
Figure S12. Cyclic voltammograms for native MBH, MBH^{C19G} and MBH^{C120G} immobilized on bare and coated PGE electrodes. page S15
Figure S13. Analysis of the MBH variants by chronoamperometry coupled with surface-enhanced infrared absorption (SEIRA) spectroscopy. page S17
Figure S14. SEIRA spectra of the MBH variants taken before and after chronoamperometry. page S20
Table S1: H_2 oxidation activity and protein yield of the purified MBH variants. page S21
Table S2. Data collection and refinement statistics of MBH cluster variants. page S22
Table S3. CO and CN stretching frequencies of [NiFe] active site redox species observed in the MBH variants. page S24
Supplementary References page S25

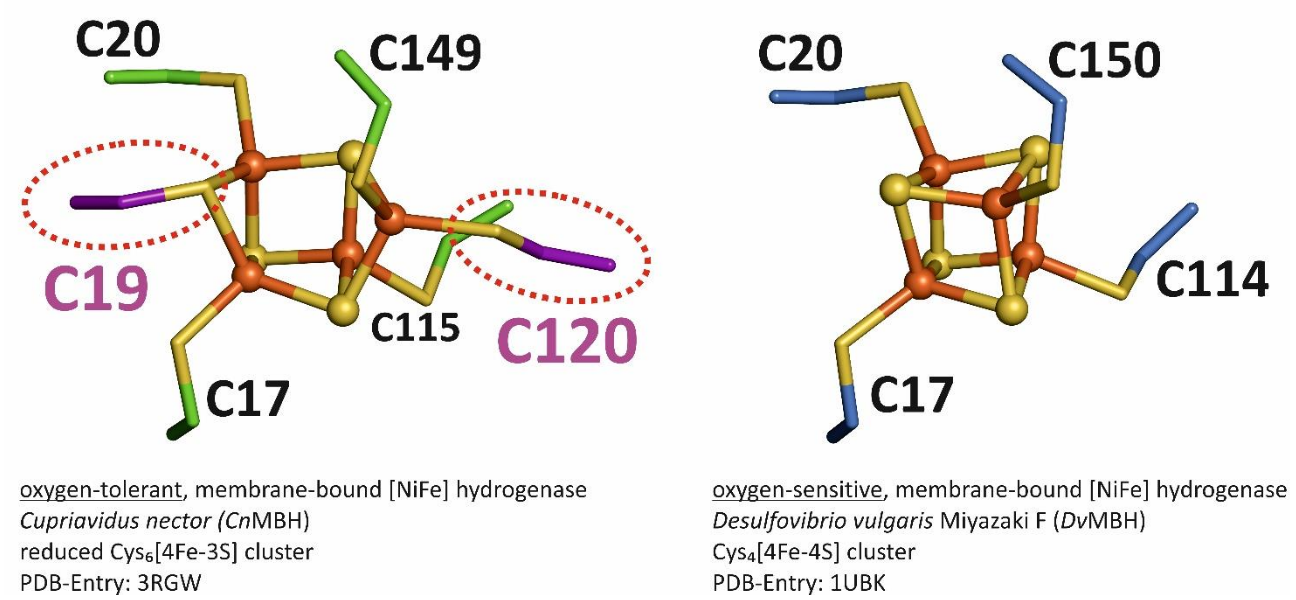

Figure S1. Proximal Fe-S clusters of oxygen-tolerant and oxygen-sensitive [NiFe]-hydrogenases. The two proximal clusters of the oxygen-tolerant MBH from *Cupriavidus necator* (left) and the oxygen-sensitive *Dv*MBH from *Desulfovibrio vulgaris* (right) are shown in ball/stick representation. Iron ions and sulfur atoms are presented as spheres in orange and yellow, respectively. The coordinating cysteines are depicted as sticks in green and blue for MBH and *Dv*MBH, respectively, with the two additional cluster coordinating cysteine Cys19 and Cys120 of MBH in purple. The thiol group of Cys19 occupies the former position of the fourth sulfur atom in the cube-shaped [4Fe-4S] cluster, leading to the formation of the [4Fe-3S] cluster. The Cys120 stabilizes the open, trapezoidal shape of the cluster.

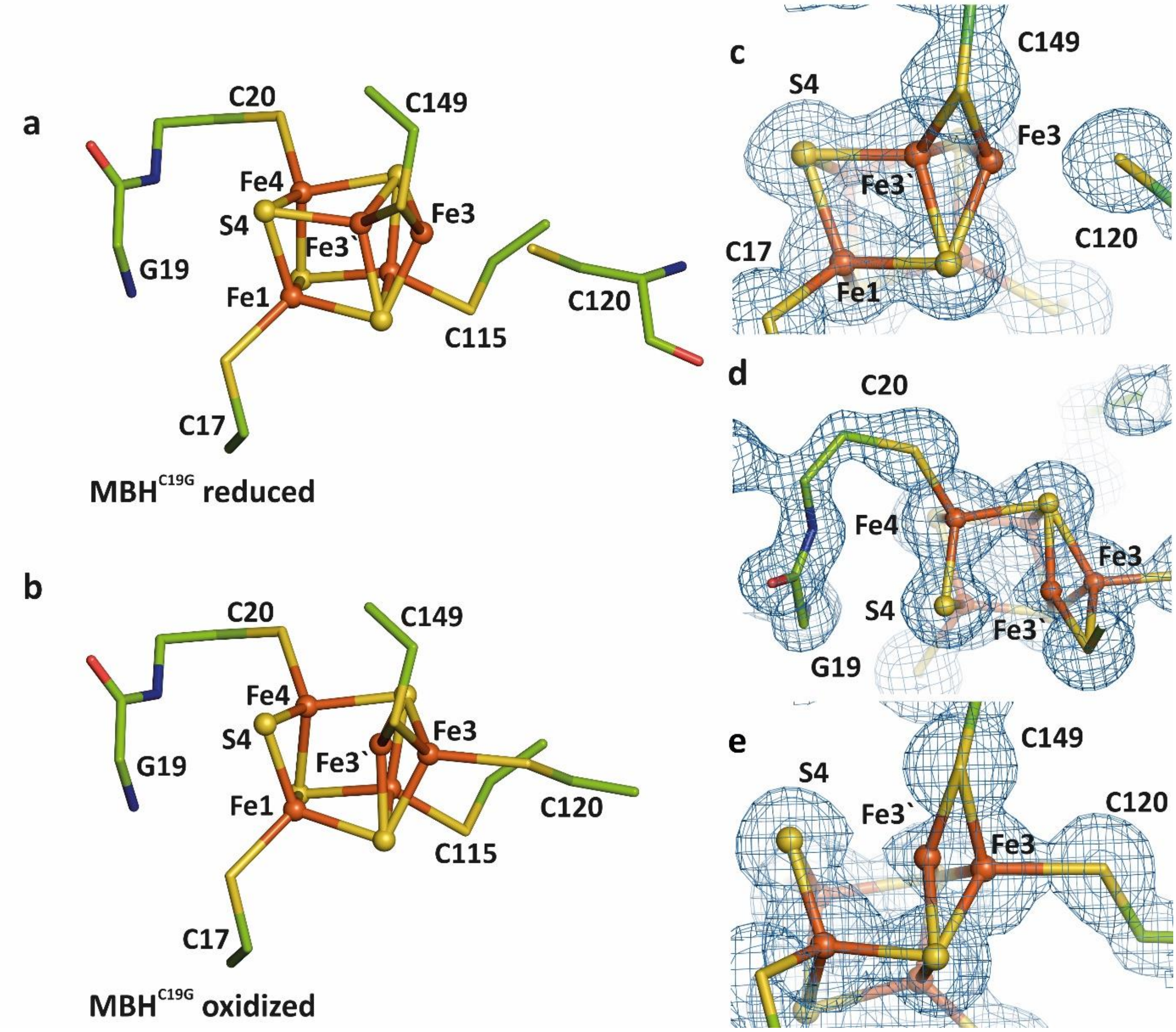

Figure S2. Proximal Cys_5[4Fe-4S] cluster structures in reduced and oxidized MBH^{C19G}. Panel (a) shows the structural model of the Cys_5[4Fe-4S] cluster of MBH^{C19G} in the oxidized state under H_2-reducing conditions and panel (b) that of the super-oxidized state under oxidizing conditions. Panel (c) shows the 2mFo-DFc electron density map (blue mesh contoured at 1.2 σ) of MBH^{C19G} in the oxidized state showing Fe3 in a double conformation (Fe3 and Fe3` position). Panel (d) shows the corresponding electron density map of MBH^{C19G} in the super-oxidized state. Note, that Fe4 is not shifted toward the backbone nitrogen of Cys20. A fraction of Fe3 forms a covalent bond with Cys120, as shown in panel (e). Color codes of the ball and stick models: green, backbone carbons; blue, nitrogen; red, oxygen; yellow, sulfur; orange, iron.

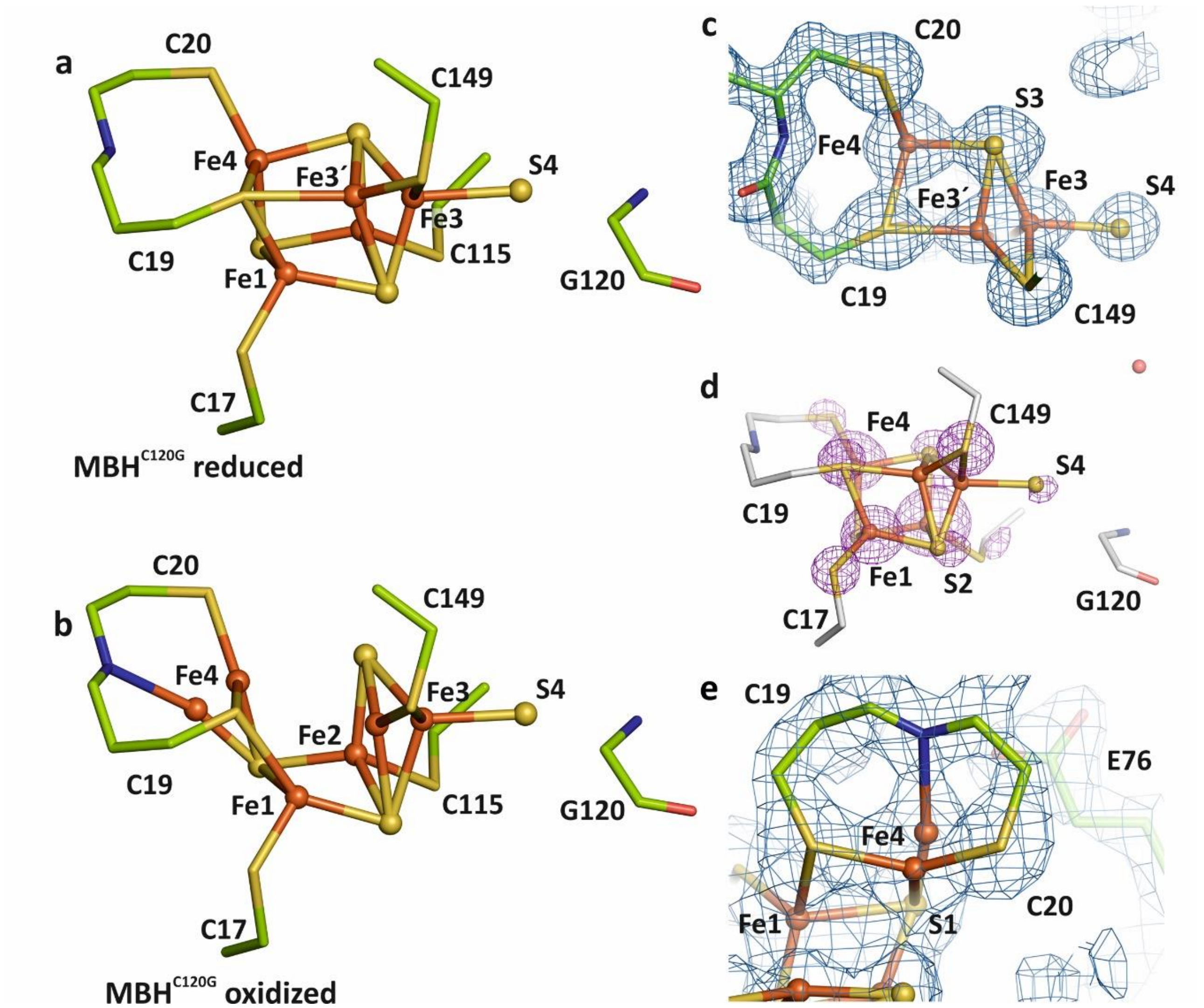

Figure S3. Proximal Cys_5[4Fe-4S] cluster structures in reduced and oxidized MBH^{C120G}. Panel (a) shows the structural model of the Cys_5[4Fe-4S] cluster *of* MBH^{C120G} in the reduced state and panel (b) that of the oxidized state. Panel (c) shows the 2*m*Fo-*D*Fc electron density map (blue mesh, contoured at 1.0 σ) of reduced MBH^{C120G}, showing Fe3 in a double conformation (positions Fe3 and Fe3`). A covalent bond was observed between Fe3 and an exo-sulfur. Panel (d) shows the result of the single-wavelength anomalous dispersion (SAD) experiment (purple meshes, contoured at 3.0 σ), identifying a sulfur species at the former thiol group position of Cys120. Panel (e) shows that Fe4 (ca. 50 %) of the oxidized cluster undergoes a redox-dependent shifting process and forms a bond with the backbone nitrogen of Cys20. Color codes of the ball and stick models: green, backbone carbons; blue, nitrogen; red, oxygen; yellow, sulfur; orange, iron.

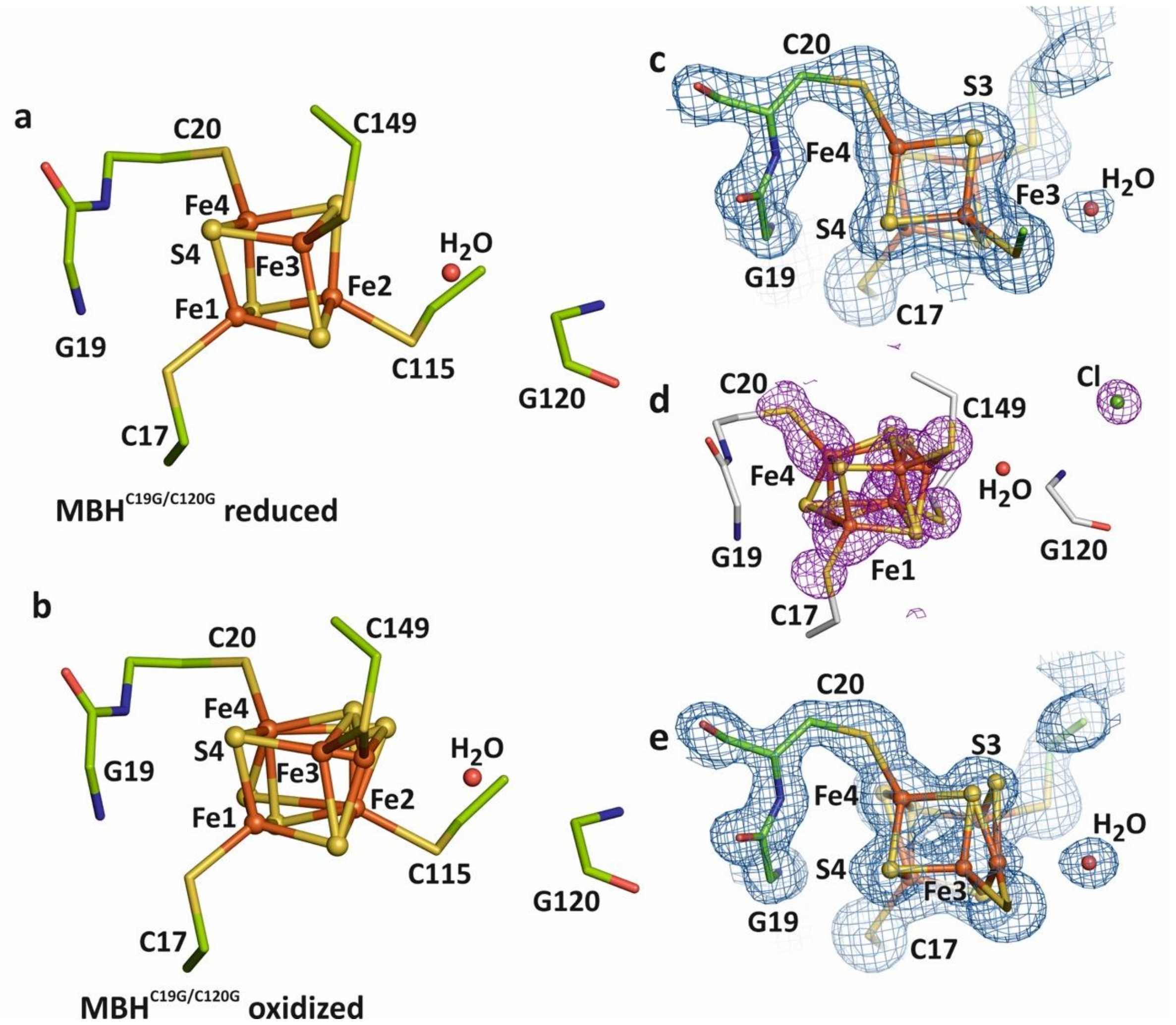

Figure S4. Proximal Cys$_4$[4Fe-4S] cluster structures in reduced and oxidized MBH$^{C19G/C120G}$. Panels (a) and (b) display the structural models of the Cys$_4$[4Fe-4S] cluster in the reduced and oxidized MBH$^{C19G/C120G}$, respectively. Panel (c) shows the 2*m*Fo-*D*Fc electron density map (blue mesh, contoured at 1.0 σ level) of the reduced cluster, indicating that the former thiolate of Cys19 is replaced with sulfide S4. Panel (d) shows the result of an SAD experiment (purple meshes, contoured at 3.0 σ), revealing a water molecule at the position of the former Cys120-derived thiolate. Panel (e) displays the 2*m*Fo-*D*Fc electron density map (blue mesh, contoured at 1.0 σ level) of the oxidized cluster, showing flexibilities of the Fe3, S1 and S3 positions.

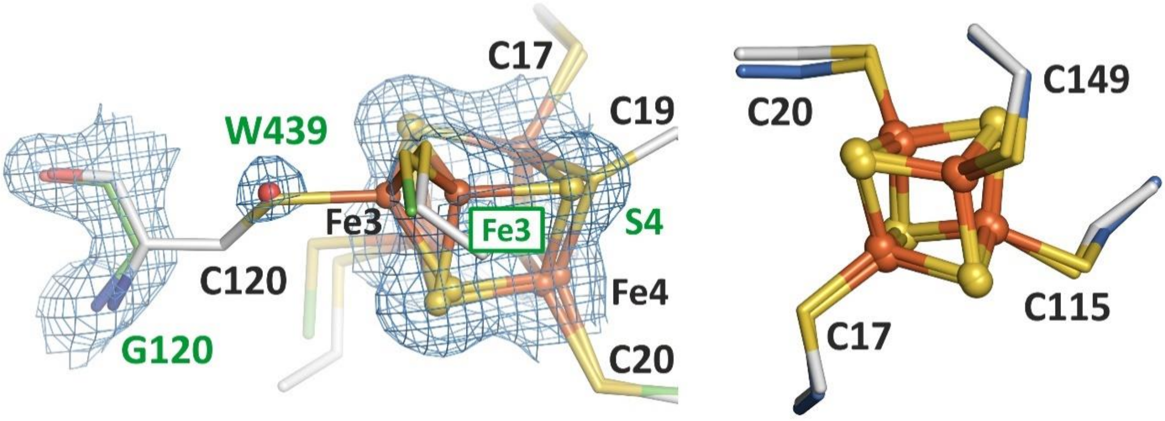

Figure S5. Comparison of the proximal Fe-S clusters of native MBH, $MBH^{C19G/C120G}$, and O_2-sensitive [NiFe]-hydrogenase. Left: Superposition of the [4Fe-4S] cluster of $MBH^{C19G/C120G}$ (backbone carbons in green) with the [4Fe-3S] cluster of native MBH (backbone carbons in white, PDB entry 3RGW). The former Cys120 sulfur atom of the [4Fe-3S] cluster is replaced by a potential water molecule (W439, red sphere). The 2*m*Fo-*D*Fc electron densities of reduced $MBH^{C19G/C120G}$ are shown as blue meshes. Right: Superposition of the reduced [4Fe-4S] cluster of $MBH^{C19G/C120G}$ (backbone carbons in white) with that of the O_2-sensitive hydrogenase from *Desulfovibrio vulgaris* (PDB entry 1UBK, backbone carbons in blue), confirming a typical cubic conformation. The canonical cysteines are Cys17, Cys20, Cys115 and Cys149 (MBH nomenclature). All clusters are shown in ball/stick representation with sulfur (S) and iron (Fe) ions as yellow and orange spheres, respectively.

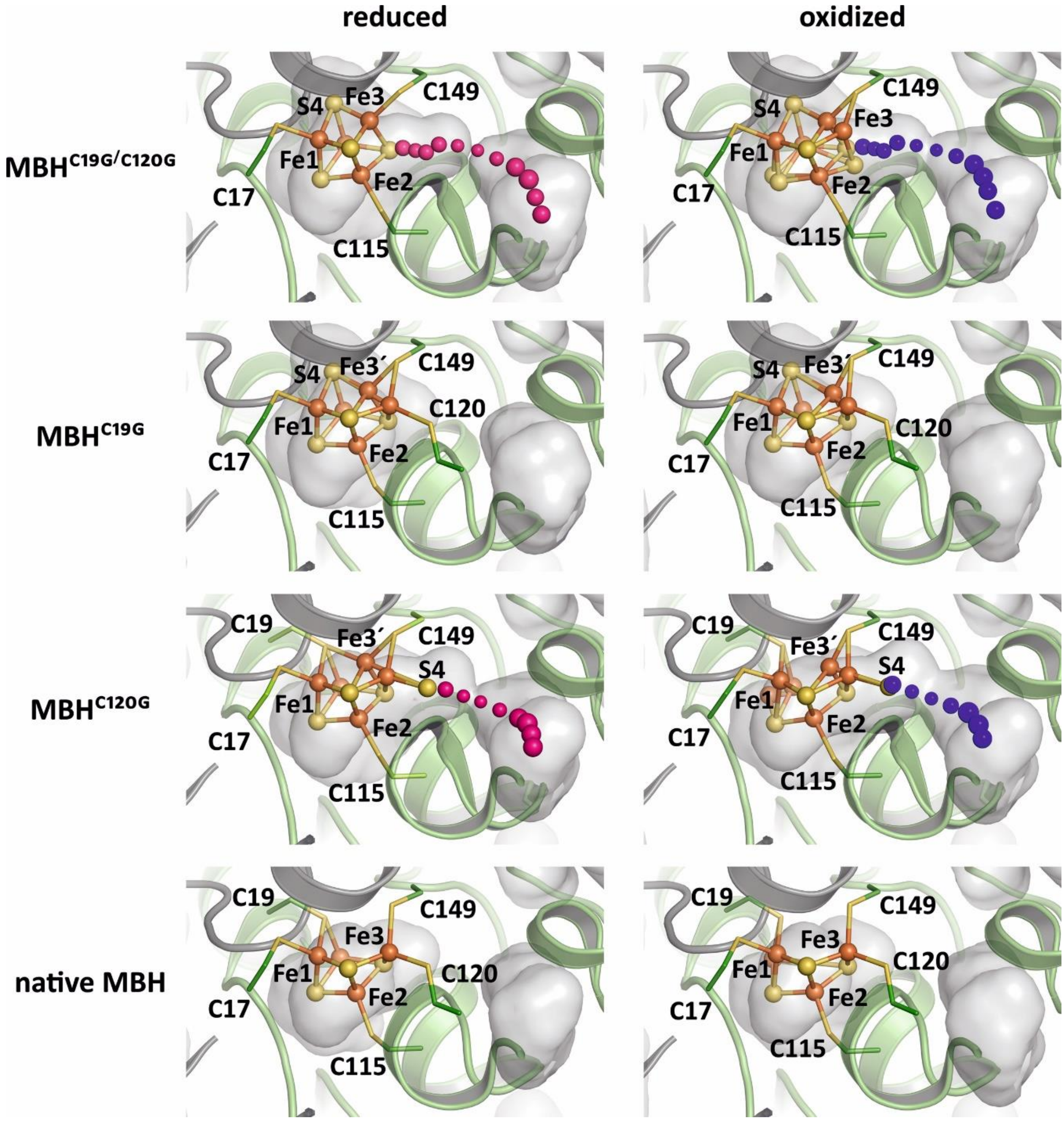

Figure S6. Solvent accessibility of the proximal Fe-S cluster in the MBH variants. The solvent accessibility was calculated with the program *CAVER 3.0*,[1] and the proximal cluster was used as the initial starting point. The substitution of Cys120 with glycine allows water molecules (purple and blue spheres) to reach the proximal cluster via a tunnel that connects the protein surface with the cluster. The diameters of the corresponding tunnel bottlenecks in the reduced/oxidized structures of $MBH^{C19G/C120G}$ and MBH^{C120G} were calculated with 1.24/1.22 Å and 1.25/1.30 Å, respectively. In case of MBH^{C19G} and native MBH, the proximal clusters cannot be accessed by water because of the bulky Cys120. The cluster atoms and the coordinating amino acids are displayed in ball and stick representation. The protein backbone is shown in cartoon representation. Cavities are illustrated with *PyMOL*.[2]

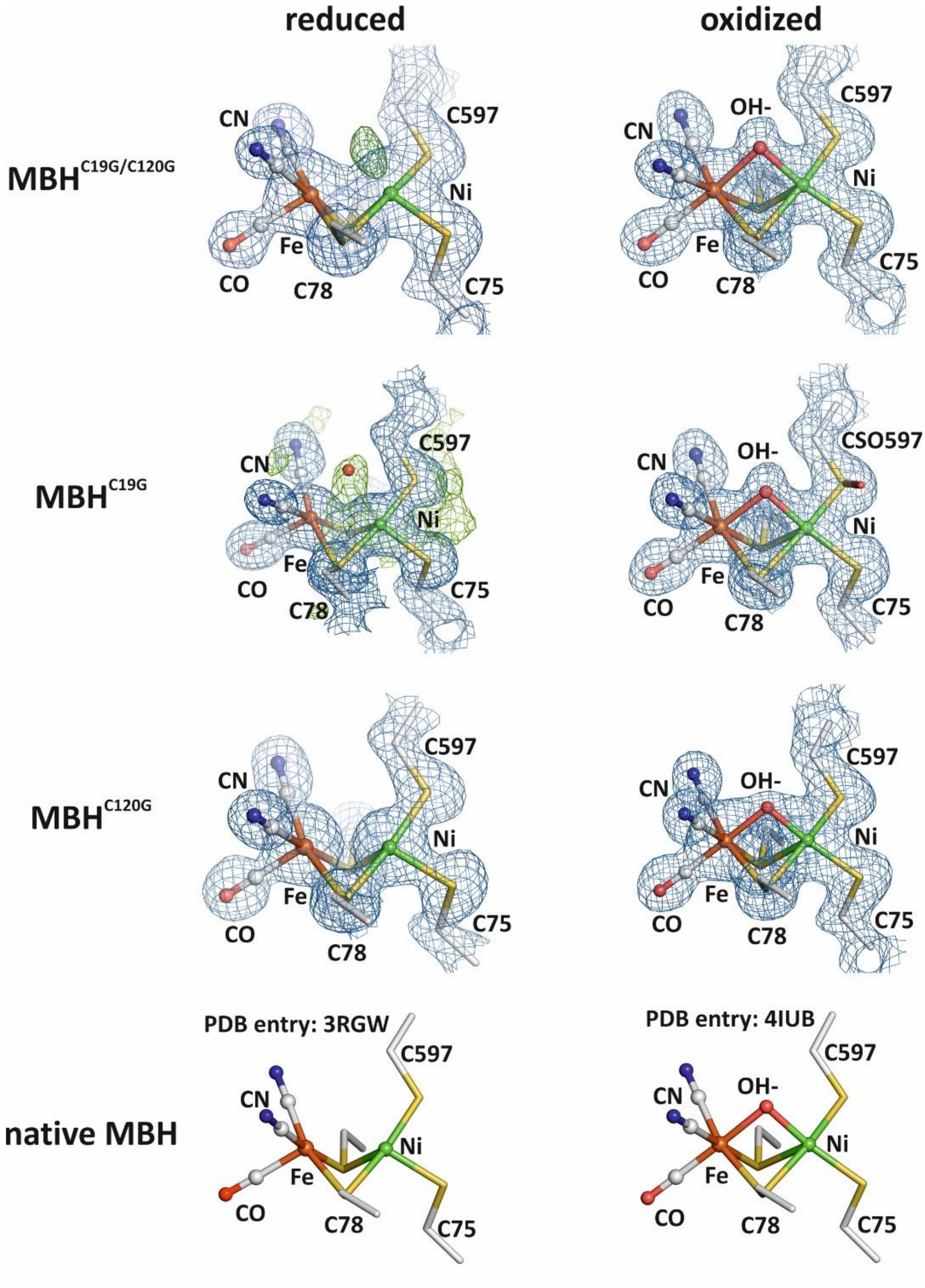

Figure S7. [NiFe]-active site structures of reduced and oxidized MBH variants. The [NiFe] active site is shown as ball/stick representation with the nickel (Ni), iron (Fe), sulfur (S), carbon (C), oxygen (O) and nitrogen (N) as green, orange, yellow, white, red and blue spheres, respectively. Electron densities are represented as blue (2*m*Fo-*D*Fc) or green (*m*Fo-*D*Fc) meshes, which were contoured at the 1.0 σ and 3.0 σ level, respectively. Additional positive *m*Fo-*D*Fc difference density was found in the reduced $MBH^{C19G/C120G}$ and MBH^{C19G} variants and interpreted as oxygen species. The corresponding occupancies were below 10 % and 40 – 70 % in case of $MBH^{C19G/C120G}$ and MBH^{C19G}, respectively. The Ni-Fe distances were generally 2.6 and 2.9 Å for the reduced and oxidized states, respectively. Only in case of reduced MBH^{C19G}, the metal-metal distance was slightly larger (2.7 Å).

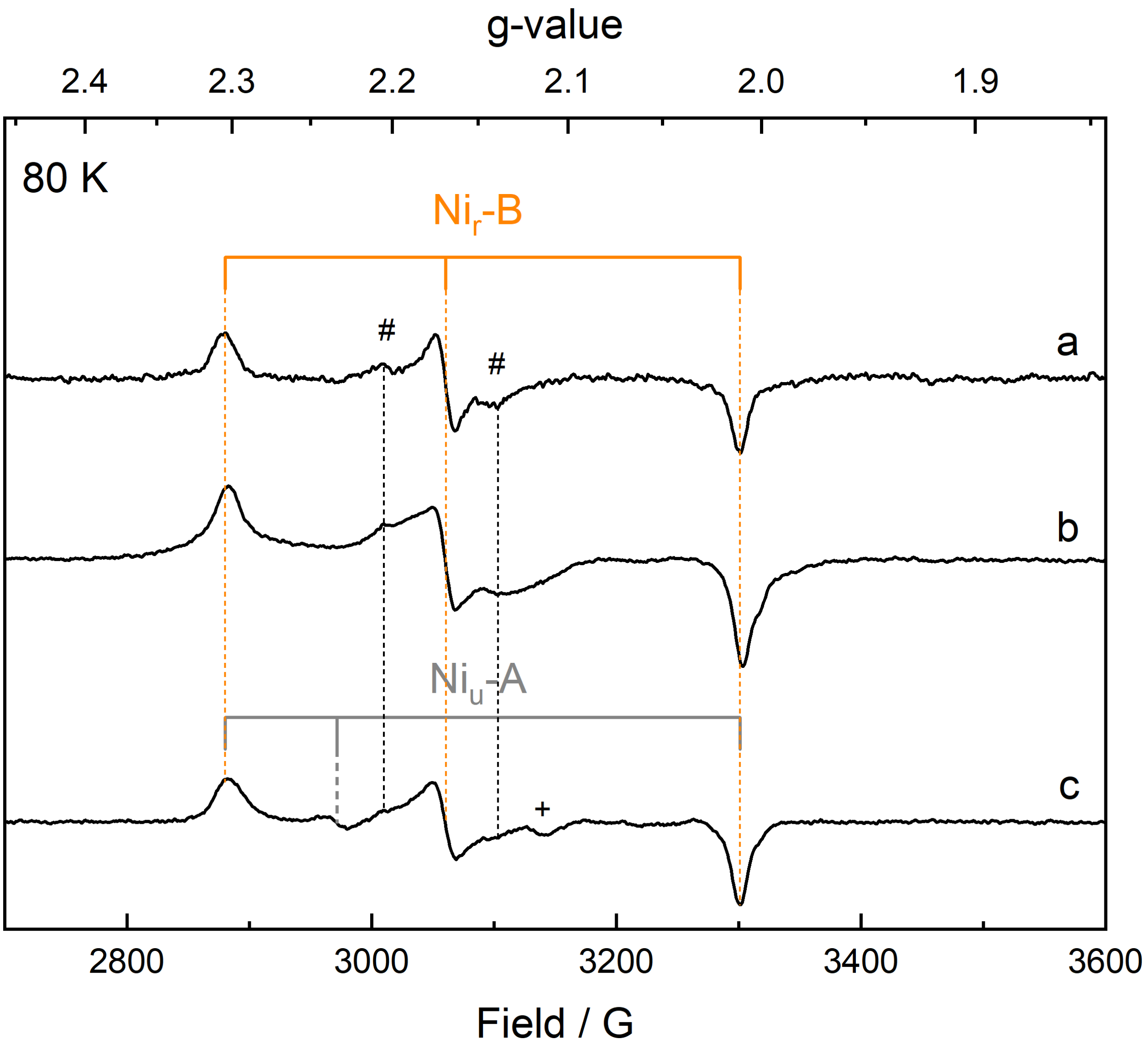

Figure S8. EPR spectra of oxidized native MBH, MBH^{C19G}, and MBH^{C120G} measured at 80 K. The spectra of (a), native MBH (b), MBH^{C19G}, and (c), MBH^{C120G} are dominated by the rhombic signal characteristic for the Ni$_r$-B state (orange, g_x = 2.30, g_y = 2.17, g_z = 2.01). Traces of additional signals (marked by #) represent so far unknown active site species. In MBH^{C120G} traces of Ni$_u$-A (grey, g_x = 2.30, g_y = 2.23, g_z = 2.01) and another species (denoted with +) were observed.

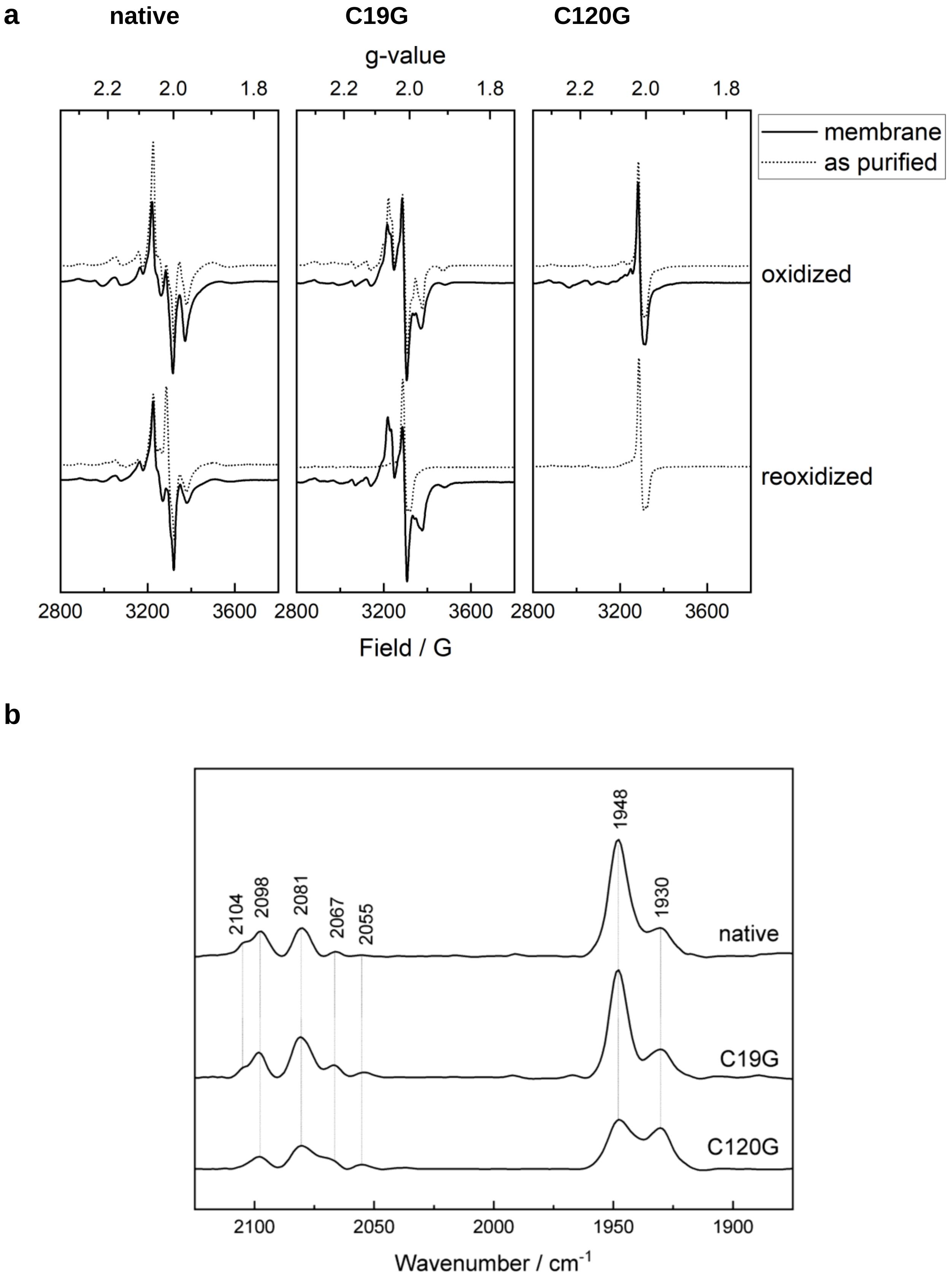

Figure S9. EPR and IR spectra of re-oxidized samples of native MBH, MBH^{C19G} and MBH^{C120G}. (Continued on the next page)

Samples of H_2-reduced MBH variants were exposed to air, leading to the re-oxidation of the enzymes.

(a) EPR spectra of native MBH (left), MBH^{C19G} (middle), and MBH^{C120G} (right) in purified heterodimeric form (dashed lines) and membrane-bound (solid lines) form. The upper and lower spectra are derived from oxidized and air re-oxidized samples. All spectra were recorded at 20 K. The spectra of oxidized samples are normalized to the signal intensity of the respective solubilized sample. The EPR spectra of oxidized native MBH and MBH^{C19G} exhibit a complex coupled signal in the region of g = 2.5-1.9, which is characteristic for the paramagnetic proximal cluster interacting with both the active site in Ni_r-B state the medial $[3Fe\text{-}4S]^+$ cluster. Upon reduction and subsequent re-oxidation with air, the complex EPR spectrum remained almost unchanged for the membrane-bound MBH samples, indicating full reversibility, when the MBH is still connected with its redox partners in the cytoplasmic membrane. Re-oxidation of the purified MBH samples, however, led to a considerable simplification of the spectra, which exhibited mainly uncoupled Ni_r-B (g_x = 2.30, g_y = 2.17, g_z = 2.01) and medial $[3Fe\text{-}4S]^+$ cluster ($g_\perp$ = 2.017; $g_\parallel$ = 2.001) signals. The EPR spectra of oxidized and re-oxidized purified MBH^{C120G} as well as oxidized membrane-bound MBH^{C120G} exhibit a very similar spectroscopic signature, comprising signals of uncoupled Ni_r-B and the medial $[3Fe\text{-}4S]^+$ cluster. The low content of MBH^{C120G} in the cytoplasmic membrane prevented its analysis after re-oxidation.

(b) IR spectra of re-oxidized purified native MBH (top), MBH^{C19G} (middle) and MBH^{C120G} (bottom). Re-oxidation of purified native MBH and MBH^{C19G} samples resulted in the recovery of mainly the Ni_r-B state (~80%), with a characteristic CO stretching vibration at 1948 cm^{-1}. In addition, a second species (~20%), with a CO absorption band at 1930 cm^{-1} was observed, which is indicative for the irreversibly inactive Ni_{ia}-S state of the active site. In case of the MBH^{C120G} variant, the signal intensity of the Ni_{ia}-S-related CO band was almost as large as that of the Ni_r-B species.

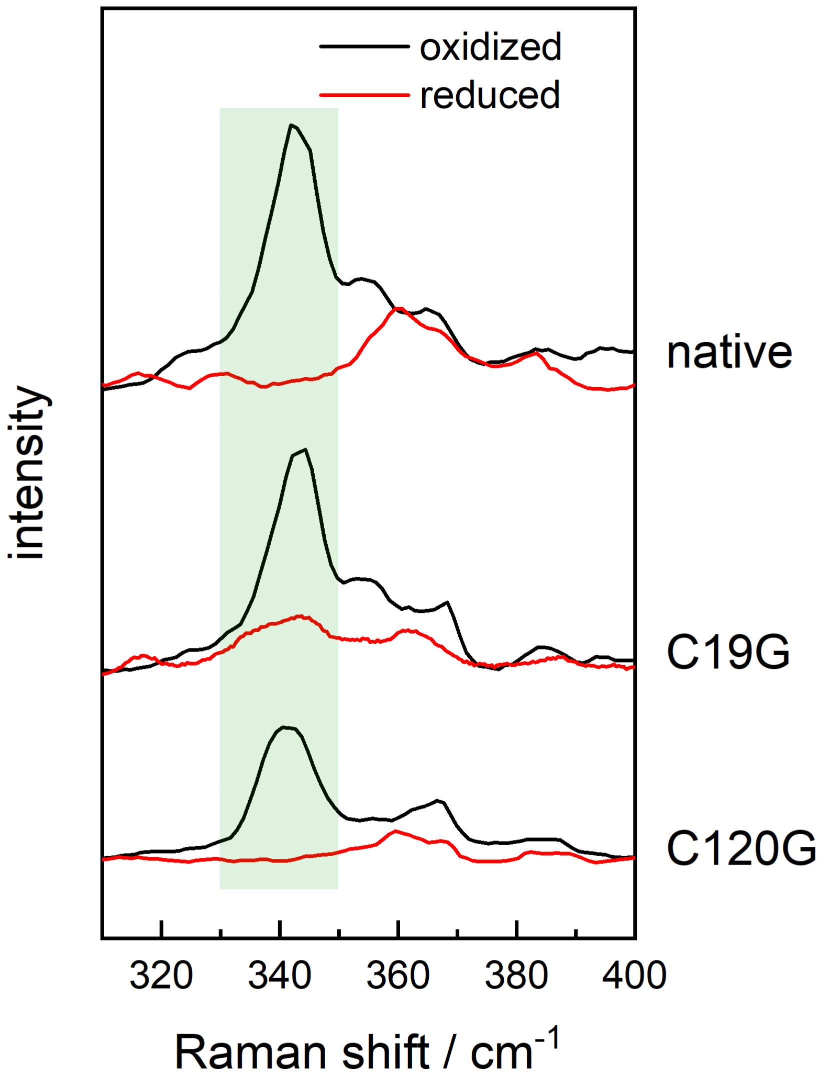

Figure S10. Resonance Raman (RR) spectra of single crystals of oxidized and reduced native MBH, MBH^{C19G} and MBH^{C120G}. Spectra acquisition was accomplished with an excitation wavelength of 458 nm from an Ar^+ laser of 1 mW power. All spectra were normalized to the phenylalanine mode at 1004 cm^{-1}. The spectra of all three oxidized MBH variants are dominated by a broad band around 340 cm^{-1} (green box) that is characteristic for oxidized iron-sulfur clusters.[3,4] The various sub-structures of the proximal cluster of the MBH^{C120G} variant (Fig. 2) might be the reason for slight shifts and variations of these Fe-S modes in the corresponding MBH^{C120G} spectrum. The weak features in the range between 355 and 375 cm^{-1} are presumably related to metal-sulfur vibrations of the active site.[3,5] Upon reduction, the intensity of the main 340 cm^{-1} band decreased considerably, which can be explained by a different electronic structure of the reduced cluster when compared to its oxidized counterpart. This, in turn, resulted in a diminished resonance enhancement.[4,6] While the band(s) around 340 cm^{-1} vanish completely in case of native MBH and MBH^{C120G}, the spectrum of reduced MBH^{C19G} still exhibits residual intensity in this range. This is in line with the presence of an HIPIP-like [4Fe-4S] cluster species that undergoes a 3^+-to-2^+ transition, as opposed to standard ([4Fe-4S]) clusters mediating a 2^+-to-1^+ transition.

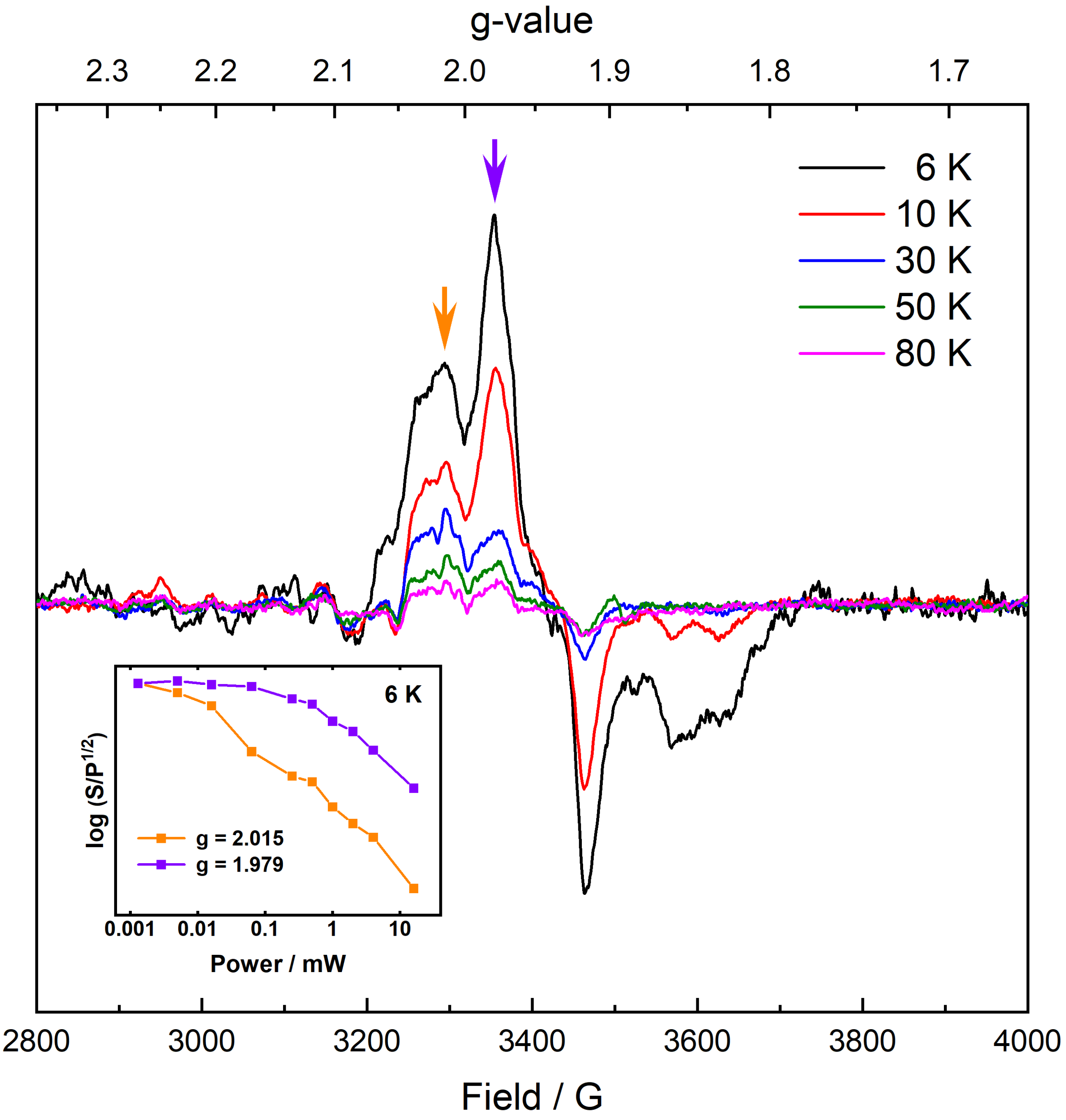

Figure S11. Temperature and microwave power dependence of the EPR signals of H_2-reduced MBH^{C120G}. With increasing temperature, the sharp signal at g = 1.979, marked by a purple arrow, decreased more strongly than the broad species at g = 2.015 (orange arrow) at a fixed microwave power of 1 mW. The two signals also exhibit a different signal (normalized to the maximum intensity) saturation with varying microwave power at a fixed temperature of 6 K (inset). This suggests the presence of at least two different electronic/structural variants of the reduced Fe-S cluster with different relaxation behaviors, which is consistent with the crystal structure data (Fig. 2c).

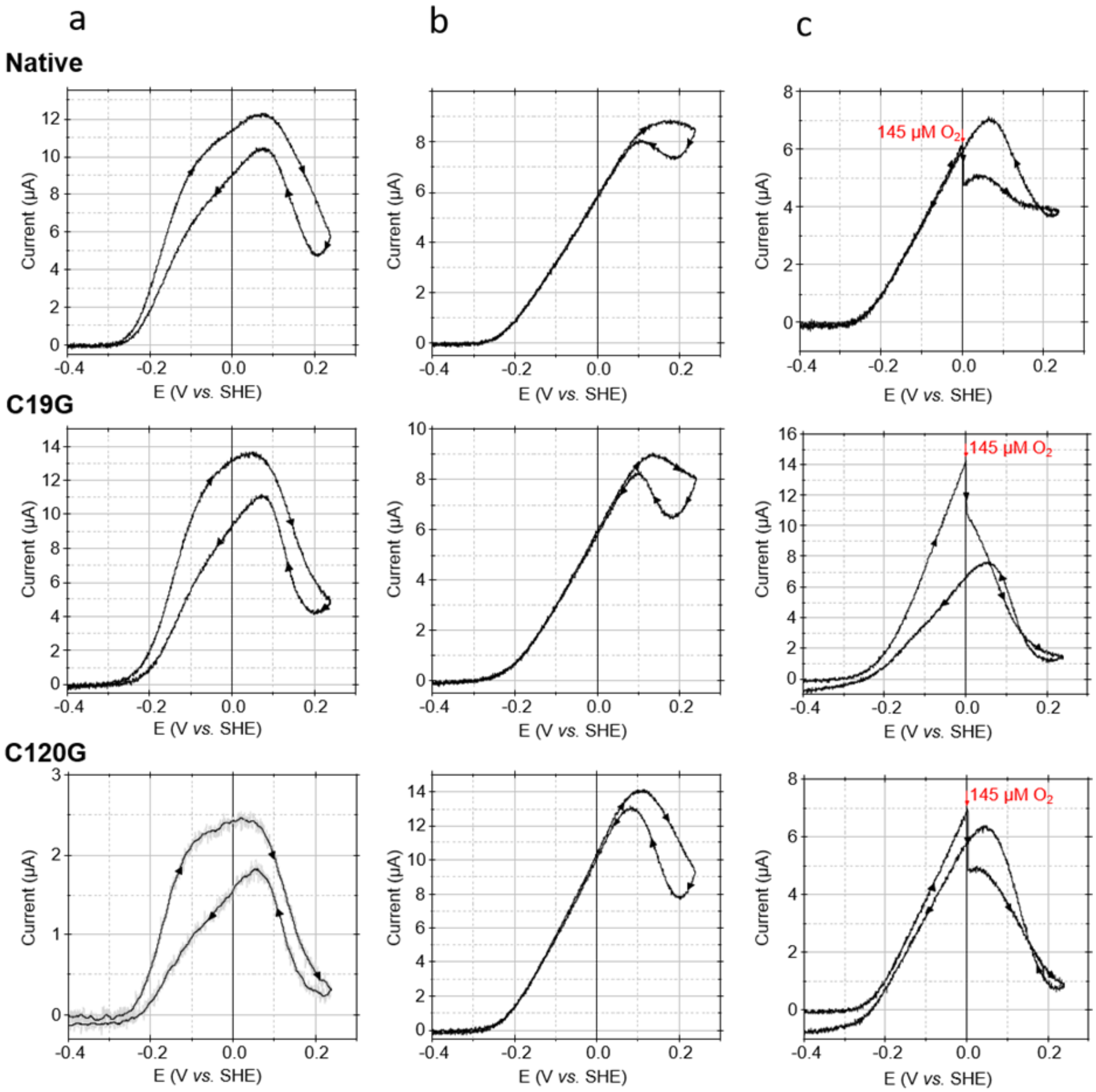

Figure S12. Cyclic voltammograms for native MBH, MBH^{C19G} and MBH^{C120G} immobilized on bare and coated PGE electrodes. (a) The purified proteins were immobilized onto pyrolytic graphite electrodes (PGE), and the electrode potential was cycled at 0.5 mVs^{-1} between -0.4 and 0.25 V (all potentials are given versus the standard hydrogen electrode) in the presence of 606 µM H_2 (80 %). All three proteins required an overpotential of approx. 80 mV to oxidize H_2, which is typical for O_2-tolerant membrane-bound [NiFe]-hydrogenases,[7] but apparently unrelated to the presence of the [4Fe-3S] cluster.[8] The H_2 oxidation current increased until a potential of approx. 50 mV was reached and then decreased because of oxidative inactivation, which is supposed to correlate with anaerobic Ni_r-B formation. The current increased again when the potential was poised back to negative potentials and reached a maximum (at approx. 50 mV), (continued on the next page).

before it started to decrease again as a result of the declining driving force. No significant difference in the voltammograms was observed for the MBH variants. The voltammograms of all three proteins, however, showed remarkable discrepancies in the currents measured in the forward and the reverse scan at 0 V, indicating either film loss or irreversible enzyme inactivation in the course of the experiment. This made it essentially impossible to distinguish between specific effects based on the amino acid exchanges and general stability effects. Therefore, an improved immobilization strategy was used to minimize current loss ([9]). (b) A PGE was first coated with multi-walled carbon nanotubes (MWNT), which were subsequently treated with 1-pyrenebutyric acid (Py). Upon chemical activation, the carboxylic groups of Py formed covalent bonds to surface lysines of the MBH derivatives. This immobilization technique enhanced the protein film stability and the electronic contact dramatically. While protein films of native MBH and the MBH^{C19G} protein showed similar cyclic voltammograms in the presence of H_2 under otherwise anoxic conditions, the MBH^{C120G} variant displayed a somewhat stronger oxidative inactivation at high potentials. (c) The enhanced protein film stability allowed for investigation of the effect of short O_2 pulses on the activity. At a potential of 0 V, which does not support direct O_2 reduction by the electrode, O_2-saturated buffer was added to a final concentration of 145 µM, and the O_2 was then rapidly removed by flushing with the initial gas mixture (80 % O_2, 20 % N_2). Indeed, O_2 caused an immediate drop in activity (20 – 30 %) for all three MBH variants (20% for C19G, 30 % for C120G). After flushing (finished after a few seconds), native MBH (clearly) and MBH^{C120G} (slightly) gained back activity even on the oxidative sweep (up to approx. 50 mV), while the activity of MBH^{C19G} dropped continuously, until the potential was raised again on the return sweep. However, at 0 V, the activity of MBH^{C19G} reached less than 50% of the initial current, indicating the formation of inactive species as a result of the incubation with O_2. The same treatment led to a loss of only about 10% of the initial activity of MBH^{C120G}, while the native MBH variant showed no activity loss on the return scan.

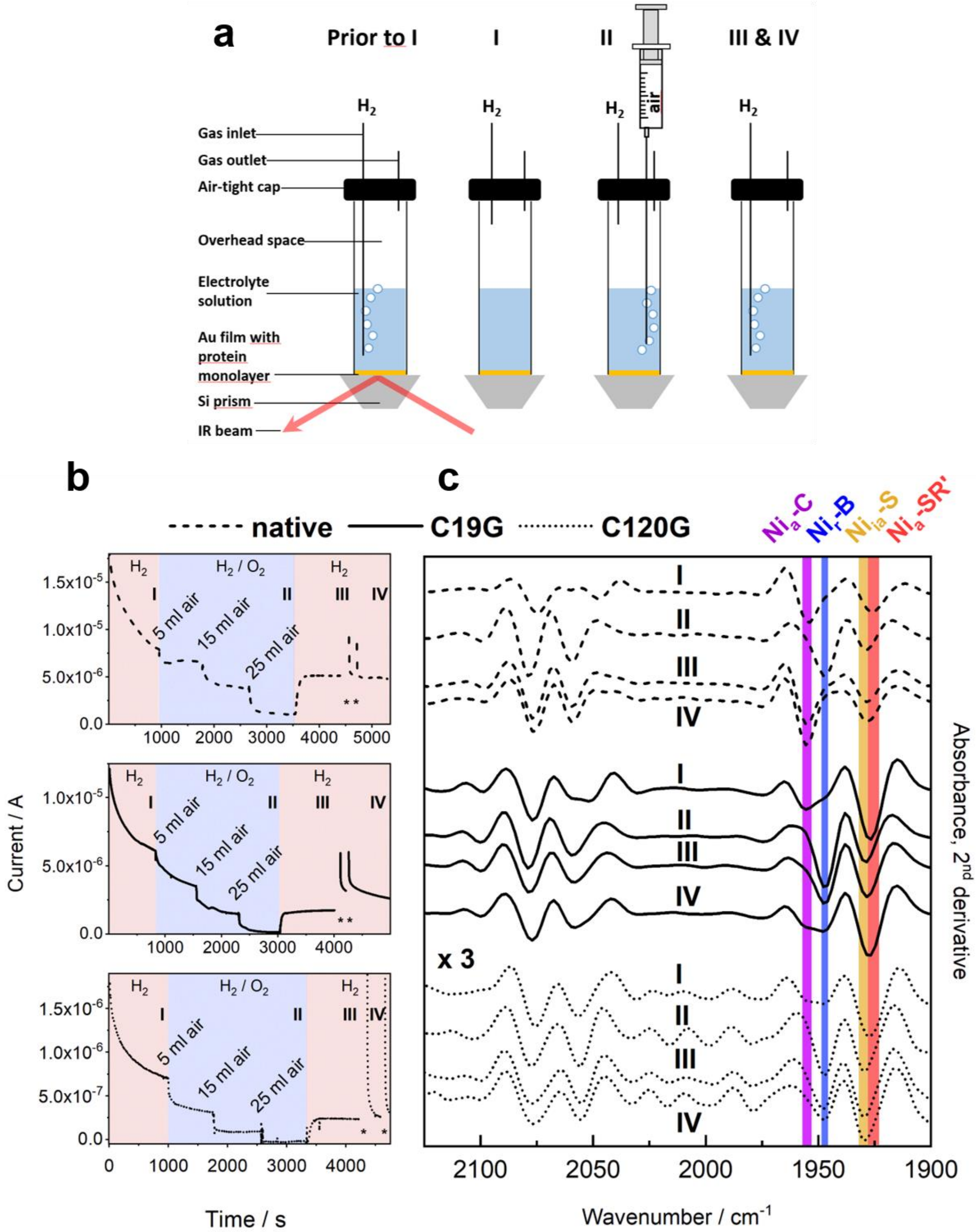

Figure S13. Analysis of the MBH variants by chronoamperometry coupled with surface-enhanced infrared absorption (SEIRA) spectroscopy. Panel (a): The MBH variants were immobilized onto SAM-modified, SEIRA-compatible gold electrodes chemically deposited on top of a silicon prism (continued on the next page).

The enzyme-covered gold-electrode was covered with 3 mL of electrolyte solution (50 mM potassium phosphate buffer, pH 5.5). The actual experiment was composed of four different phases (I-IV). Prior to phase I, the electrolyte was bubbled with pure H_2 gas to achieve full saturation with H_2 and to remove any residual oxygen. Phase I was initialized by pulling the needle out of the buffer solution (without stopping the gas flow) and adjusting the electrode potential to 0 mV vs. SHE. While maintaining the H_2 gas flow, phase II comprised the injection of three different volumes (5 mL, 15 mL, and 25 mL) of air at different time points into the electrolyte. Due to constant gassing with H_2, which is much lighter than the constituents of air, the injected air could not escape from the cell, resulting in apparently constant O_2 concentrations in the electrolyte solution. In the beginning of phase III, the H_2-releasing needle was re-inserted into the electrolyte solution to flush the remaining O_2. Upon O_2 removal, phase IV was started by applying two low-potential pulses at –400 mV after which the potential was set back to 0 mV.

Panel (b) displays the current traces representing the H_2 oxidation activities of native MBH (top, dashed line), MBH^{C19G} (middle, solid line), and MBH^{C120G} (bottom, dotted line) during the four phases of the experiment. Phase I lasted until the current reached a kind of plateau. The air injections in phase II, led to distinct current decreases. In case of native MBH and MBH^{C120G}, the current reached a constant level after each air injection. This was less obvious for MBH^{C19G}, which, however, also showed a stepwise drop in current. As the electrochemical SEIRA setup was different from that of the chronoamperometry shown in Fig. 4, O_2 concentrations and the resulting current traces of the MBH variants were not identical in the two experiments. For instance, the MBH^{C19G} variant showed a slower inactivation in the SEIRA setup, which led to a stepwise current decrease, as also observed for native MBH and MBH^{C120G}.

Notably, just partial reactivation of the MBH^{C19G} variant was obtained upon flushing the O_2 in phase III, and the H_2 oxidation current increased by applying low-potential pulses in phase IV. This behavior was observed in both electrochemical setups, and it differed from native MBH and MBH^{C120G}, whose currents were recovered just by removing O_2. Low-potential pulses led to an only negligible further current increase for MBH^{C120G} and no current increase for native MBH.

Panel (c) shows the second derivatives of the SEIRA spectra in the region characteristic for the CO absorptions. The spectra were recorded at the end of each phase, marked by a black circle in panel (b). The spectra recorded *in-situ* during catalysis under pure H_2 (phase I) were in line with the corresponding IR results for the solubilized MBH variants (Fig. 3a). While native MBH exhibited a mixture of mainly Ni_a-C (1957 cm^{-1}, purple bar) and Ni_a-SR′ (1926 cm^{-1}, red bar, with minor contributions of Ni_a-SR′′ at 1919 cm^{-1}), the spectra of MBH^{C19G} and MBH^{C120G} were dominated mostly by Ni_a-SR′. Notably, all MBH variants also showed significant contributions of the Ni_{ia}-S state (1930 cm^{-1}, orange bar) already after phase I. In case of native MBH and MBH^{C19G}, this inactive species increased slowly in the course of the experiment (phases I-IV). In the MBH^{C120G} variant, however, it was already dominant in phase I, further supporting the inherent instability of this protein (continued on the next page)

Treatment of the MBH variants with H_2/O_2 mixtures in phase II, led to a decrease of bands related to catalytic intermediates. The decreasing amount of Ni_a-SR' species led to a concomitant shift of the band at 1926 cm^{-1} to 1930 cm^{-1}, which is characteristic for the Ni_{ia}-S species. Furthermore, an absorption band at 1948 cm^{-1} (blue bar) gained in intensity, indicating the formation of Ni_r-B.
In conclusion, our results suggest the presence of two inactivation pathways. Exposure of all three MBH variants to O_2 (phase II) resulted in a current decrease, which correlated with the formation of the Ni_r-B state. In case of MBH^{C19G}, removal of O_2 from the solution was not sufficient to lower the amount of Ni_r-B state (indicated by residual intensity of the corresponding IR band after phase III), i.e. the enzyme stayed catalytically inactive. Partial recovery of catalytic current and the corresponding decrease of Ni_r-B was only accomplished by applying low-potential pulses. Hence, the apparent O_2 intolerance ascribed to the MBH^{C19G} variant seems to result from a kinetically hindered reactivation of enzyme species residing in the Ni_r-B state. The second inactivation process relates to the formation of the Ni_{ia}-S species, which continuously increased during the entire procedure for all three variants (even under reductive conditions as indicated by the blue shift of the peak maximum from phase I to III or IV towards 1930 cm^{-1}).

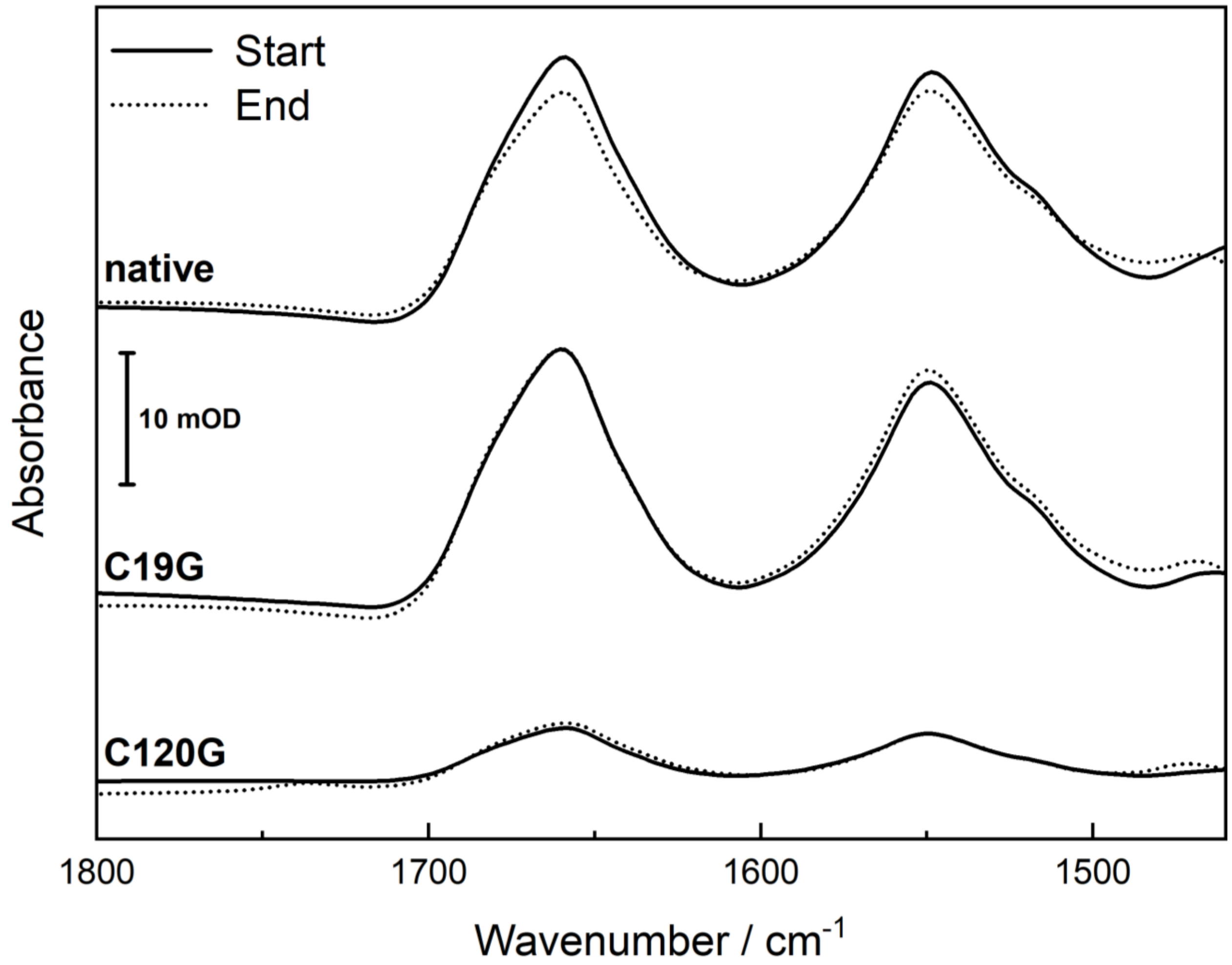

Figure S14. SEIRA spectra of the MBH variants taken before and after chronoamperometry. The spectra were recorded prior to phase I (solid lines), and after phase IV (dotted line) of the experiment described in Fig. S13. The intensity and shape of the amide I (1665 cm^{-1}) and amide II (1550 cm^{-1}) bands did not change significantly, indicating conformational stability of the protein on the SAM-modified gold electrode. Protein desorption was not observed. The lower overall band intensities for MBH^{C120G} compared to native MBH and MBH^{C19G} indicate poor immobilization, consistent with the lower current observed during chronoamperometry (Fig. S13).

Table S1 H_2 oxidation activity and protein yield of the purified MBH variants.

MBH variant	Specific activity[a] U/mg	Yield[b] µg
native MBH	140 ± 10.8[c]	425 ± 75
MBH^{C19G}	74.6 ± 5.8	584 ± 93
MBH^{C120G}	17.5 ± 7.5	35 ± 5
MBH$^{C19G/C120G}$	72.8 ± 11.7	199 ± 38

[a] H_2-dependent methylene blue reduction (1 U/mg corresponds to 1 µmol H_2 oxidized per min and per mg of protein)
[b] Protein per gram (wet weight) of cells
[c] Adopted from ref[10]

Table S2 Data collection and refinement statistics of MBH cluster variants.

	MBH^{C19G} reduced	MBH^{C19G} as-isolated	MBH^{C120G} reduced	MBH^{C120G} as-isolated
	***PDB accession 8POX**	***PDB accession 8POW**	***PDB accession 8POZ**	***PDB accession 8POY**
**Data collection**	ESRF, ID29	ESRF, ID29	ESRF, ID29	ESRF, ID29
**Space group**	$P2_12_12_1$	$P2_12_12_1$	$P2_12_12_1$	$P2_12_12_1$
**Cell dimensions *a*, *b*, *c* (Å)**	73.208 95.688 119.840	72.526 95.523 118.868	73.415 95.751 120.597	73.169 95.633 119.927
**α,β,γ (°)**	90.0, 90.0, 90.0	90.0, 90.0, 90.0	90.0, 90.0, 90.0	90.0, 90.0, 90.0
**Resolution (Å) †**	47.84 - 1.60	45.97-1.61	47.88 - 1.65	47.82-1.93
	†(1.69 - 1.60)	(1.70 - 1.61)	(1.74 - 1.65)	(2.03 - 1.93)
**R_{merge}†**	0.08 (0.64)	0.1 (0.68)	0.1 (0.59)	0.13 (0.53)
**$I/σ(I)$ †**	9.5 (2.1)	8.7 (2.3)	10.3 (2.5)	8.3 (2.5)
***CC1/2* †**	100 (88.1)	99.9 (70.6)	99.9 (92.6)	99.9 (86.1)
**Completeness (%) †**	99.9 (99.9)	99.9 (100)	98.2 (96.2)	99.9 (99.8)
**Redundancy †**	4.4 (4.4)	5.5 (5.5)	5.6 (5.6)	5.5 (5.4)
**Wilson B factor ($Å^2$)**	18.3	18.6	11.8	21.8
**Refinement**				
**Resolution (Å)**	1.60	1.61	1.65	1.93
**No. Reflections**	105721	101881	95636	60380
**R_{work} / R_{free} (%)**	12.7 / 15.6	13.7 / 16.3	14.1 / 17.2	15.3 / 18.4
**No. atoms / residues**				
**Protein (MBH)**	7139/869	6992/861	7015/869	6818 / 863
**Large subunit (HoxG)**	4956/599	4839/597	4823/599	4740 / 598
	(3-244 / 247-603)	(3-245 / 250-603)	(2-244 / 248-603)	(3-244 / 248-603)
**Small subunit (HoxK)**	2183/270 (5-274)	2152/264 (5-268)	2192/270 (5-274)	2078 / 265 (5-269)
**Others:**	684	640	858	488
**Fe-S clusters**	24 / 3	24 / 3	24 / 3	25 / 3
**[NiFe]-active site**	9 / 1	9 / 1	8 / 1	9 / 1
**Water**	641 / 640	603 / 599	822 / 813	509 / 502
**Cl^-**	2 / 1	3 / 3	3 / 2	2 / 1
**PO_4^{3-}**	1 / 1	0	0	0
**Mg^{2+}**	1 / 1	1 / 1	1 / 1	1 / 1
**Na^+**	3 / 3	0	0	0
**Mean B-factor (all atoms; $Å^2$)**	26.1	25.2	18.5	28.6
**R.m.s. ‡ deviations**				
**Bond lengths (Å)**	0.007	0.008	0.006	0.007
**Bond angles (°)**	1.086	1.254	1.141	1.04
**Ramachandran plot§**				
**% most favoured**	97.9	97.9	98.4	98.2
**Allowed**	2.1	2.1	1.6	1.8
**Disallowed**	0	0	0	0

Table S2 (continued) Data collection and refinement statistics of MBH cluster variants.

	**MBH$^{C19G/C120G}$ reduced**	**MBH^{C19GC120G} as-isolated**	**MBH^{C19GC120G} as-isolated anomalous data**	**MBH^{C120G} reduced anomalous data**
	***PDB accession 8POV**	***PDB accession 8POU**		
**Data collection**	BESSY, BL 14.1	ESRF, ID29	ESRF, ID29	ESRF, ID29
**Space group**	$P2_12_12_1$	$P2_12_12_1$	$P2_12_12_1$	$P2_12_12_1$
**Cell dimensions *a*, *b*, *c* (Å)**	73.201 95.506 118.978	73.231 95.740 120.005	73.344 95.821 120.308	73.502 95.897 120.765
**α,β,γ (°)**	90.0, 90.0, 90.0	90.0, 90.0, 90.0	90.0, 90.0, 90.0	90.0, 90.0, 90.0
**Resolution (Å) †**	34.18 - 1.92	47.87 - 1.65	47.91 - 2.11	50.0 - 2.4
	† (2.02 - 1.92)	(1.74 - 1.65)	(2.22 - 2.11)	(2.53 - 2.40)
**R_{merge} †**	0.09 (0.53)	0.07 (0.57)	0.062 (0.273)	0.098 (0.359)
***I/σ(I)* †**	9.9 (2.1)	11.7 (2.5)	26.2 (6.5)	19.5 (7.2)
***CC1/2* †**	100 (94)	100 (89.4)		
**Completeness (%) †**	100 (100)	99.7 (98.8)	97.6 (86.1)	99.6 (97.6)
**Redundancy †**	4.9 (5.0)	4.8 (4.8)	6.6 (4.6)	6.8 (6.6)
**Wilson B factor (Å^2)**	25.3	22.3	22.8	17.6
**Refinement**				
**Resolution (Å)**	1.92	1.65		
**No. Reflections**	60918	96315		
**R_{work} / R_{free} (%)**	16.6 / 19.9	13.2 / 16.1		
**No. atoms / residues**				
**Protein (MBH)**	6918 / 867	6928 / 868		
**Large subunit (HoxG)**	4782 / 598	4796 / 599		
	(3-245 / 249-603)	(3-244 / 247-603)		
**Small subunit (HoxK)**	2136 / 269 (5-273)	2132 / 269 (5-273)		
**Others:**	581	722		
**Fe-S clusters**	23 / 3	26 / 3		
**[NiFe]-active site**	8 / 1	9 / 1		
**Water**	550 / 531	692 / 667		
**Cl^-**	0	3 / 3		
**PO_4^{3-}**	0	0		
**Mg^{2+}**	1 / 1	1 / 1		
**Na^+**	0	0		
**Mean B-factor (Å^2)**	29.7	28.3		
**R.m.s. ‡ deviations**				
**Bond lengths (Å)**	0.007	0.008		
**Bond angles (°)**	1.076	1.178		
**Ramachandran plot§**				
**% most favoured**	98.0	98.5		
**Allowed**	2.0	1.5		
**Disallowed**	0	0		

* Number of crystals for data set: 1; † highest resolution shell is shown in parenthesis; ‡ R.m.s, root mean square; § as defined in the program RAMPAGE ([4])

Table S3. CO and CN stretching frequencies of [NiFe] active site redox species observed in the MBH variants. [11, 12, 13]

State	ν(CO) / cm^{-1}	ν(CN) / cm^{-1}	
Ni_r-B	1948	2080	2097
Ni_r-S	1934/6	2075	2093
$Ni_{u/r}$-S	1943	2082	2104
Ni_a-C	1957	2072	2096
Ni-L	1899	2040	2065
Ni_a-SR	1945	2068	2085
Ni_a-SR’	1925/6	2049	2072
Ni_a-SR’’	1919	2047	2072
Ni_{ia}-S	1930	2055	2067

Supplementary References

1. E. Chovancova, A. Pavelka, P. Benes, O. Strnad, J. Brezovsky, B. Kozlikova, A. Gora, V. Sustr, M. Klvana, P. Medek, L. Biedermannova, J. Sochor and J. Damborsky. CAVER 3.0: A tool for the analysis of transport pathways in dynamic protein structures. *PLoS Comput. Biol.* 2012, **8**, 23–30.
2. The PyMOL Molecular Graphics System, Version 2.5.5 Schrödinger, LLC.
3. E. Siebert, Y. Rippers, S. Frielingsdorf, J. Fritsch, A. Schmidt, J. Kalms, S. Katz, O. Lenz, P. Scheerer, L. Paasche, V. Pelmenschikov, U. Kuhlmann, M. A. Mroginski, I. Zebger and P. Hildebrandt. Resonance Raman spectroscopic analysis of the [NiFe] active site and the proximal [4Fe-3S] cluster of an O_2-tolerant membrane-bound hydrogenase in the crystalline state. *J. Phys. Chem. B*, 2015, **119**, 13785–13796.
4. S. Todorovic and M. Teixeira. Resonance Raman spectroscopy of Fe–S proteins and their redox properties. *J. Biol. Inorg. Chem.*, 2018, **23**, 647–661.
5. M. Horch, J. Schoknecht, M. A. Mroginski, O. Lenz, P. Hildebrandt and I. Zebger. Resonance Raman spectroscopy on [NiFe] hydrogenase provides structural insights into catalytic intermediates and reactions. *J. Am. Chem. Soc.*, 2014, **136**, 9870–9873.
6. F. Siebert and P. Hildebrandt. *Vibrational Spectroscopy in Life Science*. Wiley-VCH GmbH, Weinheim, Berlin
7. K. A Vincent, J. A. Cracknell, O. Lenz, I. Zebger, B. Friedrich, F. A. Armstrong. Electrocatalytic hydrogen oxidation by an enzyme at high carbon monoxide or oxygen levels. *Proc. Natl. Acad. Sci. U. S. A.*, 2005, **102**, 16951–16954.
8. M. J. Lukey, M. M. Roessler, A. Parkin, R. M. Evans, R. A. Davies, O. Lenz, B. Friedrich, F. Sargent and F. A. Armstrong. Oxygen-tolerant [NiFe]-hydrogenases: the individual and collective importance of supernumerary cysteines at the proximal Fe-S cluster. *J. Am. Chem. Soc.*, 2011, **133**, 16881–16892.
9. S. Krishnan and F. A. Armstrong. Order-of-magnitude enhancement of an enzymatic hydrogen-air fuel cell based on pyrenyl carbon nanostructures. *Chem. Sci.*, 2012, **3**, 1015–1023.
10. S. Frielingsdorf, J. Fritsch, A.Schmidt, M. Hammer, J. Löwenstein, E. Siebert, V. Pelmenschikov, T. Jaenicke, J. Kalms, Y. Rippers, F. Lendzian, I. Zebger, C. Teutloff, M. Kaupp, R. Bittl, P. Hildebrandt, B. Friedrich, O. Lenz and P. Scheerer. Reversible [4Fe-3S] cluster morphing in an O_2-tolerant [NiFe] hydrogenase. *Nat. Chem. Biol.*, 2014, **5**, 378–85.
11. M. Saggu, I. Zebger, M. Ludwig, O. Lenz, B. Friedrich, P. Hildebrandt and F. Lendzian. Spectroscopic insights into the oxygen-tolerant membrane-associated [NiFe] hydrogenase of *Ralstonia eutropha* H16. *J. Biol. Chem.*, 2009, **284**, 16264–16276.
12. M. Saggu, M. Ludwig, B. Friedrich, P. Hildebrandt, R. Bittl, F. Lendzian, O. Lenz and I. Zebger. Impact of amino acid substitutions near the catalytic site on the spectral properties of an O_2-tolerant membrane-bound [NiFe] hydrogenase. *ChemPhysChem.*, 2010, **11**, 1215–1224.
13. J. Fritsch, E. Siebert, J. Priebe, I. Zebger, F. Lendzian, C. Teutloff, B. Friedrich and O. Lenz. Rubredoxin-related maturation factor guarantees metal cofactor integrity during aerobic biosynthesis of membrane-bound [NiFe] hydrogenase. *J. Biol. Chem.*, 2014, **289**, 7982–7993.
